# Spent Graphite from End-of-Life Lithium-Ion Batteries (LIBs) as a Promising Nanoadditive to Boost Road Pavement Performance

**DOI:** 10.3390/ma14247908

**Published:** 2021-12-20

**Authors:** Nader Nciri, Namho Kim, Namjun Cho

**Affiliations:** 1 School of Industrial Design & Architectural Engineering, Korea University of Technology & Education, 1600 Chungjeol-ro, Byeongcheon-myeon, Dongnam-gu, Cheonan 31253, Chungnam, Korea; nader.nciri@koreatech.ac.kr; 2 School of Energy, Materials, & Chemical Engineering, Korea University of Technology & Education, 1600 Chungjeol-ro, Byeongcheon-myeon, Dongnam-gu, Cheonan 31253, Chungnam, Korea; njuncho@koreatech.ac.kr

**Keywords:** asphalt binder, end-of-life lithium-ions batteries (LIBs), spent graphite, TLC-FID, FT-IR, XRD, SEM, empirical tests, DSR, MSCR

## Abstract

To take swift action towards tackling the global pollution crisis of discarded lithium-ion batteries (LIBs) while reinforcing road structures, this investigation was undertaken. The influence of various proportions of spent graphite (e.g., 5, 10, and 15 wt.% SG), harvested from end-of-life LIBs, on the performance of base AP-5 asphalt cement was studied. Multiple laboratory techniques have been employed to characterize the internal physiochemical interaction between the additive and the binder. These techniques include: elemental analysis (EA), thin-layer chromatography-flame ionization detection (TLC-FID), Fourier transform-infrared spectroscopy (FT-IR), X-ray diffraction (XRD), scanning electron microscopy (SEM), empirical test methods (e.g., penetration, softening point, viscosity, and ductility), dynamic shear rheometer (DSR), and multiple stress-creep recovery (MSCR). Prior to aging, SARA analysis demonstrated that the incremental SG addition into the AP-5 bitumen reduced the contents of saturates, aromatics, and resins, and increased the proportion of asphaltenes. After aging, the saturated and aromatic hydrocarbons kept decreasing; however, the resins increased and the asphaltenes declined. Accordingly, this has brought a progressive shift tendency in the stable–colloidal system for all binders from sol-state towards sol-gel-state. FT-IR scan revealed that the SG has no apparent chemical interaction with the binder, and is endowed solely with filling effects. XRD diagnosis highlighted that the steady SG incorporation into the binder amplified its crystallinity; thereby boosting the thermomechanical properties of mastics. SEM imaging unveiled that the lower-dose of SG exhibited higher compatibility within the bitumen matrix; nevertheless, the intermediate/higher-doses made the binder body relatively rougher. DSR/MSCR/conventional tests indicated that when the asphalt is blended with the graphitic powder under unaged/aged conditions, it becomes stiffer, more viscous, and less cohesive; thereby rendering it more resistant to deformation but not to cracking. In summary, it is promisingly proven that the SG could be successfully used as an asphalt additive and could be beneficial for improving paving performance and mitigating the pollution caused by dead LIBs as well.

## 1. Introduction

The sudden amplification in road traffic over the last decades coupled with an inappropriate level of maintenance has regrettably engendered a serious degradation of road structures in numerous countries. To overcome this issue, various sorts of measurements could potentially be efficacious, such as adopting effective planning and design procedures, more effective inspection technology and construction techniques, and eventually much-improved and well-optimized materials.

The lifespan of a given asphalt pavement is drastically governed by the diverse properties of materials incorporated into the four layers of the road pavement structure (e.g., surface/wearing course, base course, sub-base course, and soil subgrade/roadbed) [1]. Multiple internal and external (e.g., environmental) factors may interfere with the performance of flexible courses including the characteristics of components (e.g., binder, additives, and aggregates) and the relative fraction of these compounds in the mix, and so on. Over the past few years, a wide array of materials has been assayed and proposed to be used either as additives or modifiers in bituminous mixes. These commercially available products are employed to greatly strengthen the structural and functional performance of road pavements comprise fillers, anti-stripping agents, antioxidants, extenders, polymers, and chemical modifiers, etc. [2].

Nowadays, there is a dramatic rise and great interest in the use of recycled materials or by-products as alternative eco-friendly materials into pavement construction for various specific purposes. This significant upsurge is occurring as a direct response to the shortage or depletion of the natural resources, the jeopardized environment, as well as to the urgent need of huge amounts of supplies for the building of pavements layers.

Various forms of recycled materials and/or by-products have demonstrated a great potential to serve as a promising ingredient for road pavement construction such as reclaimed asphalt pavement (RAP) [3], construction and demolition waste (C&D waste) [4], waste rocks [5], cement dust [6], waste glass [7], wood sawdust [8], rice husk and straw [9,10], plastic waste [11], crumb rubber [12], waste cooking oil and waste engine oil [13], and others. Certainly, the use of reclaimed materials may offer short- and long-term social, economic, and environmental advantages ranging between conserving natural resources and landfill space, curbing greenhouse gas emissions, minimizing energy consumption, lowering highway maintenance costs, and of course building sustainable pavements [14].

As one of the means towards achieving the aforementioned benefits, we have consistently fostered research into the recycling of waste materials within the transportation sector where it appears practicable, promising, and essential. Owing to the strong commitment towards supporting research in this field, we have successively employed a large variety of waste materials from various sources in roadway projects, including but not limited to expanded polystyrene waste [15], waste oyster shells [16], waste cooking oils [17], waste animal fats [18], and discarded chewing gums [19], etc. However, some pertinent recyclable materials such lithium-ion batteries (LIBs) have not yet been investigated. The spent LIB encloses precious and valuable recoverable fractions such as graphite carbon, which is potentially suitable for roadway related applications. For instance, it has been reported that the graphite could significantly improve the physico-rheological properties of asphalt binder, boost its aging resistance, and enhance its temperature and stripping properties [20]. Furthermore, graphite nanoplatelets (GNPs) could greatly extend the durability of bitumen pavements in cold areas, by suppressing the compaction effort and enhancing the low-temperature strength, as well as fracture attributes of asphaltaneous materials [21]. Likewise, it has been proven effectively that the addition of GNPs could improve the lubrification of binder when combined with mineral aggregates [22]. On top of that, the graphite powder could ameliorate the thermal conductivity of bitumen mixtures, thereby contributing to alleviate the pavement snow-melting issue [23].

Graphite represents actually the cutting-edge anode material for the majority of commercial LIBs. LIB is a type of rechargeable battery and is broadly used for portable electronic gadgets (e.g., smartphones, laptops, e-readers, digital cameras, gaming consoles, and torches, etc.) and electric vehicles (EVs) and is growing in popularity for medical, military, textile, and aerospace applications [24]. When referred to its counterparts, such as nickel–cadmium batteries (Ni-Cd batteries), LIB is endowed with higher energy density, requires less maintenance, and is characterized by lower self-discharge rates [25].

A lithium battery is made up of four key components. The first component is the cathode (i.e., a positive electrode) and consists of a complex lithiated matrix, encompassing diverse lithium metal oxide (LiMO_2_) materials including lithium cobalt oxide (LiCoO_2_), lithium manganese oxide (LiMn_2_O_4_), and lithium iron phosphate (LiFePO_4_). The cathode constitutes the source of the lithium ions and dictates the average voltage and capacity of the battery [26]. The second component is the graphite-based anode (i.e., a negative electrode), which allows the electric current to flow through an external circuit and when the electrochemical cell is charged, Li^+^ ions are emmagazined in the anode [27]. The third constituent is the electrolyte, which contains a combination of salts, solvents, and additives, and serves as a channel for conveying Li^+^ ions between the anode and cathode. The electrolytes can be found either in liquid form or semisolid/solid state. The liquid ones enclose lithium salts (e.g., LiPF_6_, LiBF_4_, LiN(CF_3_SO_2_)_2_, LiClO_4_, and LiBOB, etc.), which are dissolved in cyclic and linear organic carbonates (e.g., ethylene carbonate (EC), propylene carbonate (PC), ethyl methyl carbonate (EMC), dimethyl carbonate (DMC), diethyl carbonate (DEC) and their EC-based binary mixtures). The semisolid/solid state ones enclose lithium salts as the conducting salts along with high-molecular-weight polymer matrices (e.g., polyvinyl fluoride (PVF), polyethylene oxide (PEO), and polyvinylidene fluoride-co-hexafluoropropylene (PVDF-HFP), etc.) [28]. The last component is the separator, which is produced from synthetic resin such as polyethylene (PE), polypropylene (PP), or their blends, PE–PP. This polyolefin microporous film provides an ionic conduction path for the electrolyte and acts as a physical barrier that safely keeps the anode and cathode apart, thereby avoiding internal short circuiting [29].

The global LIB market size is projected to raise from USD 41.1 billion in 2021 to USD 116.6 billion by 2030, with a compounded annual growth rate (CAGR) of around 12.3% [30]. The growth in LIB market is skyrocketing due to the widespread use of consumer electronics [31] and hybrid/electric vehicles [32] over all the world, which ultimately drives up the incessant demands for LIBs. Consequently, it is foreseeable that the volume of end-of-life LIBs will also increase dramatically, considering their limited service life, typically varying between 3 and 5 years [33,34].

According to Yole’s analysts, there will be approximately 705,000 tons of spent LIBs by 2025, and by 2040 they will hit 9 million tons per annum [35]. Presently, only a tiny proportion of LIBs is relatively recycled through various costly, time-consuming, and energy-intensive approaches namely hydrometallurgical, pyrometallurgical, and direct mechanical recycling processes [36]. However, the rest is deplorably ending up in landfills, where it may take up hundreds of years to degrade, and potentially leak hazardous chemicals (e.g., toxic lithium salts, transition metals, etc.) into the soil and water, threatening human health and ecosystems [37]. Retired LIBs encompass also embedded electrochemical energy, which can severally provoke explosions and/or fires, eventually resulting in serious losses and injuries [38]. Several experts and specialists in materials and life-cycle analysis have related the global challenges in LIB reutilization/recycling to logistic concerns, technical obstacles, economic constraints, and regulatory loopholes [39].

To offer an effective support in tackling the global pollution crisis of discarded Li-ion batteries (LIBs), being wound massively up in the ground each year, this preliminary research project was especially designed to investigate the feasibility and effectiveness of using spent graphite (SG) recovered from decommissioned LIBs as a performance boosting-additive in road pavement applications. It was particularly conducted to assist potential stakeholders including but not limited to scientists, engineers, paving contractors, asphalt industries, transportation agencies, waste managers, etc., who are expressing a keen interest in exploring or widening their knowledge and understanding regarding the nature and characteristics of SG material that could abundantly be harvested from waste LIBs and appreciably implemented in pavement engineering. Additionally, this work was intendedly initiated to identify the various positive and negative impacts of SG on the attributes of bitumen cement before employing it as a cost-effective nanofiller in hot-mix asphalt (HMA) production and integrating it ultimately either into a pilot or large-scale paving projects.

## 2. Materials and Methods

### 2.1. Preparation of Spent Graphite (SG)-Asphalt Mastics

**Figure 1:**
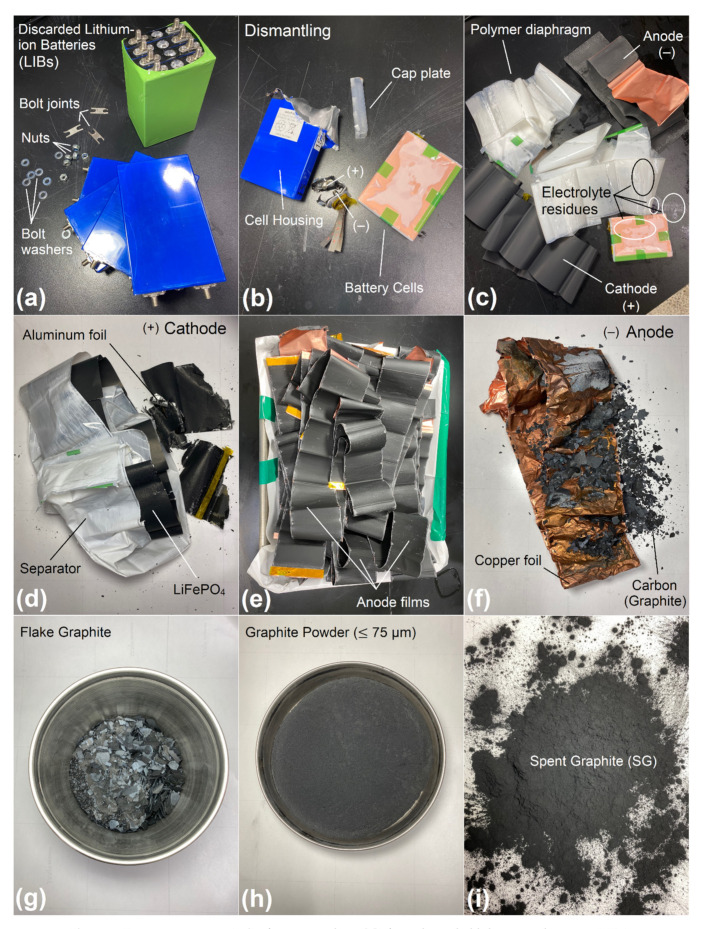
Extraction process (**a**–**i**) of spent graphite (SG) from discarded lithium-ion batteries (LIBs).

The discarded lithium-ion batteries (LIBs) were supplied by Korea Energy Lab Co., Ltd., (Suwon-si, Gyeonggi-do, Korea). Figure 1 displays a descriptive flowchart illustrating the extraction process of spent graphite (SG) from waste LIBs. To avoid the danger of short-circuit and spontaneous combustion/ignition, the spent LIBs were first fully discharged in a 5% NaCl solution for 24 h [40]. By using pliers and scissors, they were dismantled into multiple pieces according to the manual described elsewhere [41]: metallic shells, cathode rolls (i.e., fine layers of powdered lithium iron phosphate (LiFePO_4_) coated on aluminum foils), anode rolls (i.e., fine layers of powdered graphitic carbon (C) coated on copper foils), organic separator, and insulating plastic membranes. To remove residual electrolyte and moisture, the anode sheets were rinsed with deionized water and dried under vacuum oven at 60 °C for 24 h [42]. By gently crumpling and shaking off these sheets, the graphite layers can easily be peeled off from the copper foils, and the graphitic carbon flakes can be collected into a large round metallic bowl. All obtained carbonaceous samples were manually ground with a ceramic mortar and pestle into fine powder. The resulting powder was then sifted through a No. 200 (75 µm) sieve to remove any fiber or residue that may be present, and eventually conserved in hermetically-sealed metal containers, thereby enabling optimum conservation. For cost-saving and economical purposes, this recovered material will initially be used as an asphalt additive without any further pretreatment. The various chemical properties of spent graphite (SG) are synthesized in Table 1.

**Figure 2:**
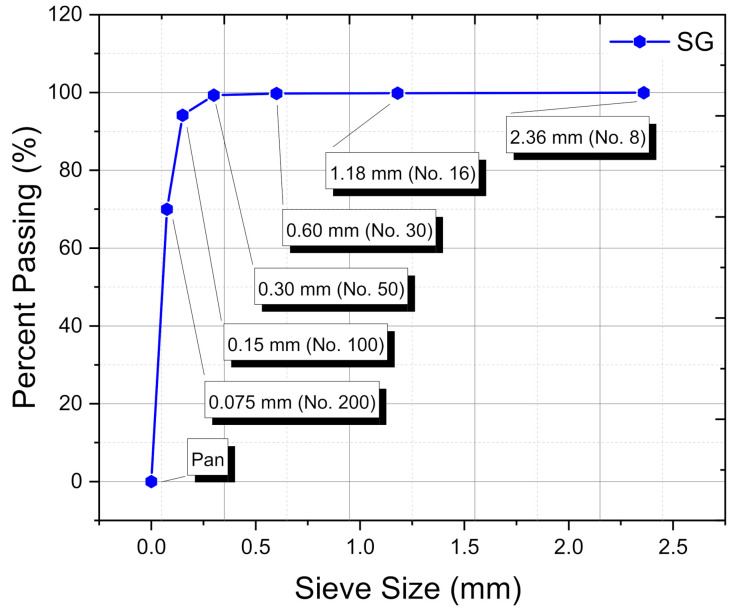
Particle size distribution (PSD) of spent graphite (SG).

The particle-size distribution (PSD) of spent graphite (SG) was determined according to ASTM D-546-17 [43] using a stacked pile of sieves aligned vertically with decreasing mesh size ranging from 8 mesh (2.36 mm) to 200 mesh (0.075 mm). One hundred grams (100 ± 0.01 g) of powdered graphite sample was agitated, vibrated, and tapped through the sieves for approximately 10 min and the quantity that remained on each screen as well as in the bottom pan was weighted and the PSD was measured and expressed in percentage (%). A typical PSD curve for SG is plotted in Figure 2.

The base AP-5 asphalt (PG 70–28) used in this investigation, was kindly procured from The Korea Federation of Ascon Industry Cooperative R&D Center (Osan-si, Gyeonggi-do, Korea). Table 2 summarizes its typical physicochemical characteristics. A L5M-A mixer (Silverson Machines Inc., East Longmeadow, MA, USA) operating at a high-shear speed of 3000 rpm along with a heating mantle (Model No. GLHMD-B100, Global Lab Co., Ltd, Siheung-si, Gyeonggi-do, Korea) running at a shear temperature of 180 °C, were utilized to fabricate the SG-asphalt admixtures [44,45,46]. The neat binder was first preheated in oven at 140 °C for 2 h to ensure a proper fluidity during mixing process. The production of dispersions was accomplished by using 1000 mL-cylindrical aluminum cans, which were filled with approximately 600 g of fluid bitumen and gradually heated to reach 175 °C [44,45,46]. At this temperature, various concentrations of powdered spent graphite (e.g., 5, 10, and 15 wt.% SG by total weight of the blend) were stepwise administered into the neat bitumen [47,48]. These lower, intermediate, and higher concentrations were intentionally selected to see clearly and closely the direct effect of SG on the physicochemical and rheological properties of base AP-5 asphalt. Additionally, it has been reported that 15 wt.% of pure graphite is optimum and could deliver promising improvement in the engineering properties of asphalt mixtures [48].

To nicely obtain homogenous mixtures, the untreated and SG-treated asphalt samples were stirred for 2 h at 180 °C [44,45,46]. When the preparation is completed, the different cans containing the asphaltaneous samples were poured, distributed into small covered metal tins, and stored at ambient temperature (ca. 25 °C) for further conditioning and testing.

### 2.2. Thin-Layer Chromatography-Flame Ionization Detection (TLC-FID)

The TLC-FID analyzer Iatroscan MK6s (Iatroscan Analyzer, Iatron Laboratories Inc., Tokyo, Japan) equipped with a metallic rack of silica rods (Type Chromarod-S5, LSI Medience Corporation, Chiyoda-ku, Tokyo, Japan) was used to investigate the effect of various contents of spent graphite (e.g., 5, 10, and 15 wt.% SG) on the composition of base AP-5 asphalt before and after aging. Before being spotted, the set of 10 chromarods (length 15.2 cm, pore diameter 60 Å, particle size 5 μm) was activated and cleaned several times by the hydrogen flame of the FID. This step was executed not only to ensure sufficient removal of undesirable residues or impurities, but also to yield accurate and reproducible data. After preparation of 2% (*w*/*v*) asphaltaneous solution in dichloromethane, 1 µL of sample solution was ejected from glass vials by means of a 5-μL Drummond Microdispenser (Drummond Scientific, Broomall, PA, USA) and carefully deposited onto the origin-points of freshly-activated chromarods. When the spotting process is accomplished, the rod holder was dried at ambient condition and sequentially sojourned in three closed-development tanks containing several solvents namely (1) *n*-hexane for 45 min (2) toluene for 17 min, and (3) dichloromethane/methanol (95/5) for 5 min. This enables the fractionation and elution of saturates, aromatics, resins, and asphaltenes (i.e., SARA generic fractions), respectively [19]. In order to diminish the sample spreading, the remaining solvents were removed after each development stage, by placing the frame in a drying oven for 3 min at 120 °C. Afterwards, the rack was mounted on the Iatroscan. The FID was set at constant air and hydrogen flow rates of 2000 mL min^−1^ and 160 mL min^−1^, respectively and the scan speed was 30 s/scan [19]. The TLC-FID data were acquired and treated using Clarity Lite Software (Futecs Co., Ltd., Sinildong-ro, Daedeok-gu, Daejeon, Korea). To ensure reproducibility and repeatability, the Iatroscan analysis was repeated six times.

The stability–compatibility of SG–bitumen mixtures before and after aging/modification was predicted by means of colloidal instability index (I_C_), which is expressed as the mass ratio of the sum of asphaltenes and its flocculants (i.e., saturates) to the sum of its solvents (i.e., aromatics) and peptizing agents (i.e., resins), using the following Equation (1) [49,50]:

(1) $$I_{C}=\frac{\left[\mathrm{Saturates}\right]+\left[\mathrm{Asphaltenes}\right]}{\left[\mathrm{Aromatics}\right]+\left[\mathrm{Resins}\right]}$$

### 2.3. Fourier-Transform Infrared Spectroscopy (FT-IR)

To probe any sort of alterations or changes occurring at the molecular structure level of the base AP-5 bitumen after its modification/aging with various proportions of spent graphite (e.g., 5, 10, and 15 wt.% SG), FT-IR was carried out. The FT-IR data were directly received by using a Hyperion 3000 FT-IR Spectrometer (Bruker Optics, Ettlinger, Germany) and adopting a wavenumber range between 4000 and 650 cm^−1^, an average of 30 scans per specimen, and a spectral resolution of 1 cm^−1^. The spent graphite (SG) powder, plain bitumen, and SG-modified bitumen specimens were scanned using thin disk of the sample blended with KBr (i.e., potassium bromide).

### 2.4. X-ray Diffraction Spectroscopy (XRD)

The XRD scan was performed to assess the impact of various doses of spent graphite (e.g., 5, 10, and 15 wt.% SG) on the microcrystallographic structure and phase of fresh-plain bitumen. The XRD patterns of the asphaltaneous samples were recorded by using a Bruker AXS D8 Advance Diffractometer (Bruker AXS GmbH D8 Advance, Karlsruhe, Germany) under the following conditions: ambient temperature 25 °C, CuKα (radiation λ 1.54005 Å, tube voltage 40 kV, tube current 40 mA), scanning range 10° ≤ 2θ ≤ 90°, scanning rate 1° min^−1^, and step size 0.05.

### 2.5. Scanning Electron Microscopy (SEM)

The influence of various proportions of spent graphite (e.g., 5, 10, and 10 wt.% SG) on the surface topography, morphology, as well as chemical composition of original base AP-5 bitumen was detailly investigated by means of JSM-6010LA Scanning electron microscopy (SEM) (JSM-6010LA, JEOL Ltd., Tokyo, Japan) combined with Energy-Dispersive X-ray Spectroscopy (EDXS). Before proceeding with SEM, the diverse bituminous specimens were fully submerged in a liquid nitrogen (LN_2_, −80 °C), and then were coated with a thin layer of gold (ca. 10 nm) using X sputter coater, to increase their electrical conductivities. The SEM micrographs were captured under the following conditions: accelerating voltage 5 kV, beam current 5 nA, working distance (WD) of 10 nm, and magnification (×3000).

### 2.6. Conventional Binder Tests (Penetration, Softening Point, Viscosity, and Ductility)

To investigate the direct effects of various contents of spent graphite (e.g., 5, 10, and 15 wt.% SG) on the physical and rheological properties of base AP-5 bitumen, several empirical lab tests were conducted in quadruplicate following numerous ASTM standards: According to ASTM D5 [51] the penetration test, which estimates the degree of softness or hardness of a given asphalt binder, was performed by means of Humboldt Mfg Electric Penetrometer (Humboldt Mfg. Co., Elgin, IL, USA). By maintaining the bitumen temperature at 25 °C, a standard 100 g-loaded needle was allowed to vertically penetrate for 5 s into the binder body, thereby measuring the penetration in 1/10 mm. Referring to ASTM D36 [52], the softening point test was carried out by using Ring and Ball Test Apparatus RKA 5 (Anton Paar GmbH, Ashland, VA, USA). This test was applied to determine the consistency of binder and gain further knowledge regarding the temperature (T_R&B_) at which will reach a specified level of viscosity. To get access to the consistency, under a load of 1 cm-diameter steel ball, a thin disk of asphalt was allowed to flow for a distance of 2.5 cm. By following the procedure described in ASTM D4402 [53] and adopting a Brookfield DV III Rheometer (Brookfield, Middleboro, MA, USA), the rotational viscosity (RV) which represents the internal friction of the bitumen was obtained. It was determined at a temperature of 135 °C by sensing the torque needed to spin a SC4-27 spindle at 20 rpm while fully submerged in 10 ± 0.5 g flowing bitumen. The ductility was carried out at 25 °C as per ASTM D113 [54]. It measures the distance (cm) to which an asphaltaneous sample will stretch in a water bath before breaking down when a standard briquette specimen of the material is pulled apart at a stretching velocity of 5 cm min^−1^.

### 2.7. Laboratory Asphalt-Aging Procedure

Before being submitted to dynamic shear rheometer (DSR) scan and other conventional laboratory test methods, the plain bitumen (i.e., base AP-5 asphalt), along with its specimens blended with several fractions of recovered graphite powder (e.g., 5, 10, and 15 wt.% SG), underwent artificial aging through rolling thin-film oven test (RTFO) and pressure aging vessel (PAV). The short-term aging RTFO was carried out according to ASTM D8272-19 [55] by placing the glass bottles containing the asphalt samples, weighting each 35 ± 0.5 g, in a rolling oven (Model CS325, James Cox & Sons, Inc., Colfax, CA, USA) operating at 163 ± 0.5 °C for 1 h and 25 min with an air flow rate of 4000 mL min^−1^. This technique was adopted to predict oxidative aging occurring generally during production, transport, and compaction processes of hot-mix asphalts. The long-term aging PAV was executed following the test method outlined in ASTM D6521-13 [56]. Briefly, the RTFO-asphalt samples weighting each 50 ± 0.5 g were poured into stainless steel pans and immediately placed in air-pressurized PAV (PAV3, Applied Test Systems LLC, Butler, PA, USA) at 2.1 ± 0.1 MPa and running at 100 °C for 20 h. This technique was designed to simulate 5–10 years of in-service aging. To eliminate air bubbles that formed during PAV-aging, the bituminous samples were degassed at 170 °C for 30 min in a VDO 81-PV2610 (i.e., vacuum degassing oven, NOVA Measurements LLC, Atlixco, Pueblo, Mexico). Lastly, the aged samples were kept hermetically sealed in metal containers for further immediate testing.

### 2.8. Dynamic Shear Rheometer (DSR) Test

To gain an in-depth knowledge and understanding regarding the impact of various amounts of spent graphite (e.g., 5, 10, and 15 wt.% SG) on the viscoelastic characteristics of base AP-5 asphalt, a Dynamic Shear Rheometer (DSR) produced by ThermoFisher (Thermo Scientific^TM^ HAAKE^TM^ MARS^TM^ Rheometer, ThermoFisher Scientific, Newington, NH, USA) was utilized. According to ASTM D7175 [57], DSR test was particularly conducted to monitor rutting at higher temperatures (e.g., 46–82 °C) and fatigue cracking at intermediate temperatures (e.g., 4–40 °C). At a loading frequency of 10 rad s^−1^ (1.59 Hz), the untreated and SG-treated asphalt samples were rheologically characterized and the results were defined in terms of stiffness or complex shear modulus |G*| and plastic deformation potential or phase angle (δ), which in turn they will be combined together to generate the so-called rutting factor (G*/sin δ) and fatigue cracking factor (G*.sin δ); decisive factors for the roadway structure durability. Depending on the testing temperature, two specimen sizes were adopted: for higher temperatures (46–82 °C), a bitumen specimen with 25-mm diameter and 1-mm thickness was used and for intermediate temperature (4–40 °C), a bitumen specimen with 8-mm diameter and 2-mm thickness.

### 2.9. Multiple Stress-Creep Recovery (MSCR) Test

To properly assess the direct influence of different proportions of spent graphite (e.g., 5, 10, and 15 wt.% SG) on the base AP-5 asphalt’s potential for permanent deformation, as well as the efficiency and rate of SG modification, MSCR test was conducted by using a Dynamic Shear Rheometer (DSR) from ThermoFisher (Thermo Scientific^TM^ HAAKE^TM^ MARS^TM^ Rheometer, ThermoFisher Scientific, Newington, NH, USA) in accordance to AASHTO T 350-19 [58]. The MSCR test was performed at 64 °C, which is the average 7-day maximum pavement design temperature in South Korea, by using a plate–plate geometry with a 1.0-mm-gap height and 25-mm diameter. This test procedure consists of two stress levels 0.1 kPa and 3.2 kPa, and at each stress level every asphalt sample was subjected to ten cycles divided between loading and recovery. The whole test lasts for only 6 min until it is completed. Before being allowed to recover for 9 s (i.e., stress-free phase or recovery), the RTFO-conditioned asphaltaneous samples were loaded for 1 s (i.e., creep). At the end of experiment, several valuable and available parameters can be issued including the non-recoverable creep compliance (*J_nr_*), known also as rutting potential index, and the percent recovery (*R%*). The *J_nr_* is defined as a measure of residual strain remained in the sample after undergoing repeated creep-recovery cycles, with respect to the total amount of stress applied. The *J_nr_* is used as an indicator for the sensitivity to stress dependence and permanent deformation. Meanwhile, the *R%* is a measure of how thoroughly the sample returns to its original form once the load has been removed. The *R%* was employed to identify the existence of stress dependence and elastic response of understudied bitumen samples. The *J_nr_*_0.1_, *J_nr_*_3.2_, ∆*J_nr_*, and *R%* were obtained by using the following Equations (2)–(5) [59,60]:

(2) $$J_{nr0.1}\left(\frac{1}{\mathrm{kPa}}\right)=\frac{1}{10}\left\{\overset{10}{\underset{n=1}{\sum }}\left(\frac{\mathrm{Non}-\mathrm{Recoverable}\mathrm{Strain}}{0.1}\right)\right\}$$

(3) $$J_{nr3.2}\left(\frac{1}{\mathrm{kPa}}\right)=\frac{1}{10}\left\{\overset{10}{\underset{n=1}{\sum }}\left(\frac{\mathrm{Non}-\mathrm{Recoverable}\mathrm{Strain}}{3.2}\right)\right\}$$

(4) $$\Delta J_{nr}\left(\%\right)=\left\{\left(\frac{J_{nr3.2}-J_{nr0.1}}{J_{nr0.1}}\right)\times 100\right\}\leq 75\%$$

(5) $$R\left(\%\right)=\frac{1}{10}\left\{\overset{10}{\underset{n=1}{\sum }}\left(\frac{\mathrm{Recoverable}\mathrm{Strain}}{\mathrm{Peak}\mathrm{Strain}}\right)\times 100\right\}$$

## 3. Results and Discussion

### 3.1. Thin-Layer Chromatography-Flame Ionization Detection (TLC-FID)

Thin-layer chromatography with flame ionization detection (TLC-FID) is a simple, fast, and reliable technique that allows to quantify and separate several bitumen specimens into distinctive component groups, commonly known as SARA (i.e., Saturates, Aromatics, Resins, and Asphaltenes) fractions according to their polarity and solubility, without preliminary precipitation of asphaltenes [61]. The TLC-FID analysis was initiated to effectively and efficiently assess the impacts of various dosages of spent graphite (e.g., 5, 10, and 15 wt.% SG) on the generic composition of neat base AP-5 asphalt before and after short-term (RTFO) and long-term (PAV) aging.

**Figure 3:**
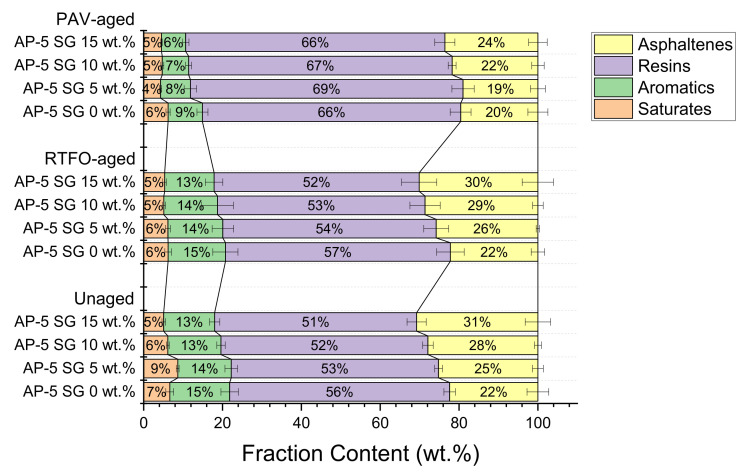
Effect of diverse doses of SG (e.g., 5, 10, 15 wt.%) on the SARA generic fractions of base AP-5 asphalt before and after RTFO and PAV aging.

As visualized in Figure 3, the plain bitumen is characterized by higher amounts of resins (55.78 ± 1.44 wt.%), lower amounts of saturates (6.63 ± 1.00 wt.%), and moderate quantities of aromatics (15.18 ± 2.20 wt.%) and asphaltenes (i.e., *n*-heptane insoluble fraction, 22.38 ± 2.75 wt.%). On the other side, the spent graphite (SG) is predominantly made up of asphaltenes with mass fraction of approximately (88.16 ± 1.02 wt.%). Whereas, the content of saturated hydrocarbons/paraffins (5.32 ± 0.82 wt.%), aromatic hydrocarbons (2.50 ± 0.22 wt.%), and polar aromatics (4.00 ± 0.57 wt.%) are negligible/inconsiderable.

Reliant on Figure 3, it can be found that the steady introduction of graphite into the unaged plain bitumen induced a slight drop in the percentages of saturates, aromatics, and resins; and eventually generated a lift in the proportion of asphaltenes. This implies that following the injection of several SG shots into the fresh binder body, its light components such as saturates and aromatics were instantaneously absorbed by the porous graphite particles; thereby leading to enriching the bitumen matrix in more polar asphaltenes molecules and in turn resulting in its viscosity increase. It should be mentioned that the additive’s particle-size distribution (PSD ≤ 0.75 µm) plays a crucial role in governing the absorption–adsorption mechanisms. Usually, the diffusion rate of oily components into smaller particle sizes is much higher than larger ones [62,63]. In other words, finer particle sizes dispose higher surface area and hence higher tendency to sorb the lighter molecular-weight fractions more than the coarser ones [62,63]. The non-polar saturates did not undergo any gross alteration due to their inert chemical character.

Preliminary screening of data plotted in Figure 3 points out schematically that the SARA-fractions distribution of all thermal-conditioned asphalt samples underwent an upward shift towards higher chemical polarity after thermal treatment, with respect to unaged asphaltaneous specimens. In this connection, laboratory oxidative aging procedures significantly affected the composition of binder as well as its chemical structure, giving rise to more polar compounds rather than non-polar compounds. For instance, the RTFO- and PAV-aged bituminous samples compounded with numerous portions of spent graphite (i.e., 5, 10, and 15 wt.% SG) exhibited virtually similar trends towards higher polarity with aging. In the course of artificial weathering, the saturate and aromatic fractions marked a decrease due to the combined effect of heat and/or modification; meanwhile, the content of resins recorded a striking growth at the expense of aromatics and asphaltenes, respectively, thereby indicating that the binder becomes harder and more brittle/viscous.

The unexpected contraction in PAV-asphaltene yields could be primarily contributed to the abundance of resin molecules, which are obviously empowered with high ability to percolate and break down the microporous structure of asphaltenes, leading to the diffusion of asphaltenes-resins particles into the eluting solvent (i.e., dichloromethane/methanol) [64]. However, further in-depth investigations are strongly suggested to comprehensively assess the unpredicted diminution of asphaltenes content during aging. The change on saturates was barely noticeable due to their lower chemical reactivity towards aging.

### 3.2. Colloidal Instability Index (I_C_)

To meticulously monitor the exact change in the degree of instability–incompatibility between spent graphite (SG) and base AP-5 asphalt before and after aging/modification, the colloidal instability index or Gaestel index (I_C_), which offers a general insight into the microscale of binder, was adopted.

Bitumen is conventionally considered as a colloidal system, in which aromatics along with resins act as asphaltene stabilizers; whereas, saturates and asphaltenes are regarded as solidoid (i.e., asphaltenes) destabilizers. The I_C_ is expressed as the ratio of the dispersed phase or unfavorable compounds [flocculants (saturated oils) + asphaltenes] to the dispersing phase or favorable compounds [peptizers (resins) + solvents (aromatic oils)] of the gas oil (i.e., saturates and aromatics) [49,50]. The lower the I_C_ value, the better the stability of asphaltenes in oil. If I_C_ ≥ 0.9, the asphaltenes are unstable in the oily-based medium and tending to precipitate or flocculate, forming an incessant–weak network. If I_C_ ≤ 0.7, the asphaltene clusters are well stable in the dispersive–oily medium, highly arranged by the resins. For I_C_ values ranging between 0.7 and 0.9, there is some sort of uncertainty regarding the stability [49,50].

**Figure 4:**
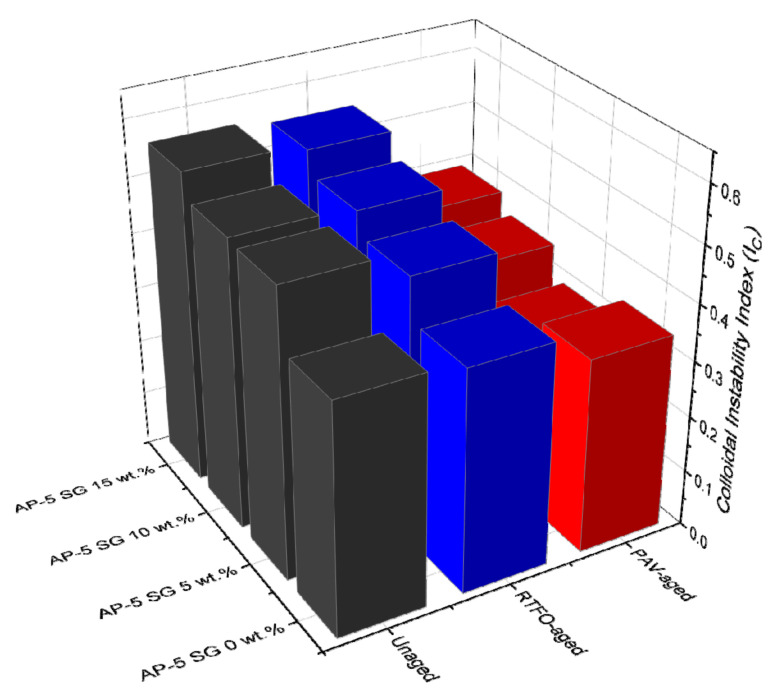
Effect of diverse doses of SG (e.g., 5, 10, 15 wt.%) on the colloidal instability index (I_C_) of base AP-5 asphalt before and after RTFO and PAV aging.

In light of data available from Figure 4, the fresh virgin-base AP-5 bitumen possesses the lowest I_C_ value (0.40) as compared to its samples modified with different percentages of SG (i.e., 5, 10, and 15 wt.%), thereby validating its stability nature. On the other hand, the I_C_ measurements showed a tendency to increase with increasing SG fraction, demonstrating a steady decrease in stability, translated seemingly by a continuous formation of asphaltenes aggregates throughout the bituminous bulk. In parallel, a gradual decrease in penetration/ductility along with an increase in softening points/viscosity were clearly witnessed at the binder level. The impact of short-term aging (RTFO) on the I_C_ change was moderate; however, was overwhelming with long-term aging (PAV), while maintaining the same evolution trend of unaged asphalt specimens. The decrease in the I_C_ values during aging cannot be attributed solely to the condensation of a large amount of naphthenic aromatics into resins but also to the conversion of some amount of asphaltenes into resins. This mechanism will certainly reinforce the interactions between the peptizing agents (i.e., resins) and the asphaltene cores, leading eventually to preventing them from detachment or debonding from the dispersive medium, and hence allowing the whole system stabilizes thermodynamically.

From the colloidal chemistry prospect, the bitumen binder is pictured as a suspensoid, in which several micelles consisting of a resinous shell and an asphaltenic core are dispersed in a continuous maltenic phase (i.e., an admixture of aromatics and saturates) [65]. Based on the equilibrium status between SARA components, the bituminous binder can be classified into three categories: (1) sol-type (i.e., mainly liquid consistency, viscous/Newtonian behavior), (2) sol-gel type (i.e., mainly solid–liquid consistency, viscoelastic behavior), and (3) gel-type (i.e., mainly solid consistency, elastic/non-Newtonian behavior) [66].

Sol-bitumina (I_C_ ≤ 0.7) are showing a relatively considerable amount of resins/oils and lower proportion of asphaltenes. The asphaltene micelles are randomly flowing in a homogenous medium [67]. This type of binder disposes inferior viscosity coupled with greater plasticity–fluidity and higher temperature susceptibility. After undergoing cracking, the sol-structure bitumina are liable to heal [67]. The unaged and aged base AP-5 asphalt spiked with various shots of spent graphite (i.e., 0, 5, 10, and 15 wt.% SG) belong to this class of binders. Through RTFO and PAV aging stages, with the buildup of resins, the suspension of asphaltenes acquires higher stability and produces smaller conglomerates.

Sol-Gel bitumina (0.7 < I_C_ < 1.2) have a satisfactory amount of asphaltene micelles and display both elastic and viscous response [67]. It has been reported that this class of binders consists of 15–25 wt.% asphaltenes, properly organized in the form of micelles, which are widely dispersed in the oily phase [67]. The increase in micelle content is directly proportional to the increase of plastic deformation and the material shows a pseudoplastic behavior. Such binders are effectively useful in road pavement construction due to their higher rheological performances [67].

Gel-bitumina (1.2 < I_C_) contain well-organized and numerous asphaltene micelles. They are commonly known with a great viscosity, and minor plasticity–fluidity associated with low-temperature vulnerability [67]. In comparison with sol-bitumina, they are structured as “gels” and possess low-kinetic dependency. This category of binders has a wide relaxation array but its phase angle is slightly related to the complex modulus deviation. Gel-bitumina are suitable to be explored as architectural-petroleum asphalts [67].

Collectively, the observations discussed throughout this article point out the efficiency and effectiveness of colloidal instability index (I_C_) in predicting the instability–incompatibility between the spent graphite (SG) and the base AP-5 asphalt; however, to add more credibility and reliability on this claim, further practical examinations (e.g., Stankiewicz plot (SP), qualitative-quantitative analysis (QQA), toluene equivalence (TE), heptane dilution (HD), spot test, and oil compatibility model (OCM), etc.) are highly required.

### 3.3. Fourier-Transform Infrared Spectroscopy (FT-IR)

The Fourier-transform infrared spectroscopy (FT-IR) scan was carried out to gain further information about the effects of supplementing different levels of spent graphite (e.g., 5, 10, and 15 wt.% SG) to the virgin base AP-5 asphalt both prior and after aging.

**Figure 5:**
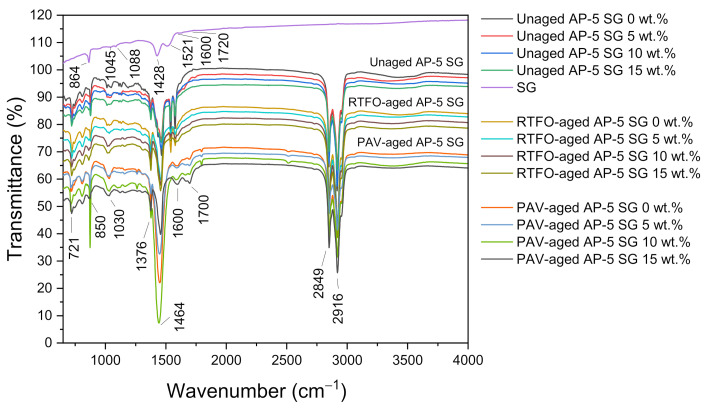
FT-IR spectra of spent graphite (SG), unmodified and base AP-5 asphalt modified with various proportions of SG (e.g., 5, 10, and 15 wt.%) before and after RTFO and PAV aging.

Figure 5 illustrates a bunch of IR spectra split between recovered graphite, unaged, short-term aged (RTFO), and long-term aged (PAV) bituminous specimens containing various SG contents.

The IR spectrum of unaged/neat bitumen (i.e., Unaged AP-5 SG 0 wt.%), including as well the IR spectrum of SG, gave rise to a weak and broad band centered around 3100–3500 cm^–1^ region, which is mainly stemmed from the stretching/bending vibrations of N–H and/or O–H functional groups. The two peaks with strong signals occurred around 2916 cm^–1^ and 2849 cm^–1^ and belong to the CH_2_ asymmetric and symmetric stretching vibrations, respectively. The C–H asymmetric bending vibrations of –(CH_2_)_n_ are detected at 1464 cm^–1^, whereas, the C–H asymmetric bending vibrations of –CH_3_ are emerged at 1376 cm^–1^. The vibration signal at 721 cm^–1^ corresponds mainly to the *in*-plane bending (i.e., rocking) of –CH_2_. Taking a closer inspection of these bands, which primarily originated from alkyl functional groups, it can arguably be said that the saturated components did not show any considerable reactivity neither towards aging nor towards SG addition, corroborating their relatively high chemical inertness.

The characteristic –C=C– stretching vibrations of aromatic rings can be perceived as a diminutive shoulder at 1600 cm^–1^ for both graphitic and asphaltaneous samples. The C–H stretches (i.e., wags) of para-, meta-, and ortho-substituted benzene rings have generated several peaks located at 850, 723, and 548 cm^–1^ (data not shown for the last band), respectively. All the peaks related to aromatic compounds were effectively affected by aging/modification and exhibited a continuous growth in intensity after thermal/SG treatment; revealing a considerable increase in aromatic compounds (i.e., more particularly asphaltenes) in the asphalt cement.

The carbonyl (C=O) peak, which usually occurs at 1700 cm^–1^, was invisible in the IR spectrum of unaged/unmodified base AP-5 asphalt; however, it becomes gradually remarkable after undergoing RTFO and PAV aging processes and additive treatment. Simultaneously, the sulfoxide (S=O) peak at 1030 cm^–1^ noted also a steady increase in intensity after exposure to artificial weathering. By broadening their bands, the aging influence was more pronounced on sulfoxide compounds than carbonyl ones; thereby revealing, their higher sensitivity to oxidation. The increase in the concentration of C=O and S=O groups translates actually a direct increase in the concentration of large polar molecules in the aged binders, resulting in an accentuated stiffness associated with more solid-like behavior and thus more susceptibility to thermal cracking. Following the 5 wt.% SG treatment, the signals of oxidation bands registered, curiously, a slight reduction, indicating that the spent graphite (SG) has imparted some anti-aging properties to the unaged/virgin bitumen.

A typical FT-IR transmission spectrum for the spent graphite (SG) is portrayed in Figure 5. No significant peaks were occurred for waste anode-graphite, which is mostly made up of carbon. The absorption peak at 1521 cm^–1^ region arouse chiefly from the –C=C– bond generated by sp^2^ hybridization. The skeletal vibrations of C–C six-membered ring forms can be depicted by the sharp peak at 1045 cm^−1^, revealing the unique sp^2^ plane structure of graphite. Meanwhile, the two peaks at 1428 and 1088 cm^−1^ are the characteristic features of the O–H bending vibrations and –C–OH stretching vibrations, respectively [68]. The existence of oxygen-containing functional groups, along with the carbonyl –C=O group emerged as a very weak band at 1720 cm^−1^, substantiates the fact that the spent graphite (SG) understudy has been already oxidized.

The Graftonite or Iron (II) Phosphate (Fe_3_(PO_4_)_2_), which may exist in negligible quantities, can be identified by the two weak bands at 1050 cm^–1^ and 979 cm^–1^, which are assigned to the symmetric and asymmetric stretching vibration modes of P–O in phosphates (–PO_4_^3–^), respectively [69]. The medium-sharp peak at 864 cm^–1^ could be created due to the stretching vibration of P–F belonging to the lithium hexafluorophosphate (LiPF_6_) electrolyte [70]. The Griceite (LiF) cannot be sensed within the IR scan range (i.e., 4000–650 cm^–1^) because it usually occurs at lower frequency vibration modes (i.e., 385 and 580 cm^−1^) [71].

After the SG administration into the fresh/virgin bitumen, all the infrared absorption bands derived from the anode-graphite of spent LIBs reported a progressive rise in signal intensity. Nevertheless, no new peaks have been produced due to the mixing of the additive with the binder; indicative evidence that the reaction was physical in nature but not chemical. Accordingly, it could be admitted that the spent graphite (SG) works as an additive and not as a modifier. However, further in-depth investigation will be required to confirm this assumption and uncover all the underlying mechanisms involved in the interfacial interaction between mineral filler and asphalt binder.

### 3.4. X-ray Diffraction Spectroscopy (XRD)

As an attempt to evaluate the impact of various portions of spent graphite (e.g., 5, 10, and 15 wt.% SG) on the microcrystalline structure and microphase of the neat base AP-5 asphalt, the X-ray diffraction spectroscopy was performed accordingly.

**Figure 6:**
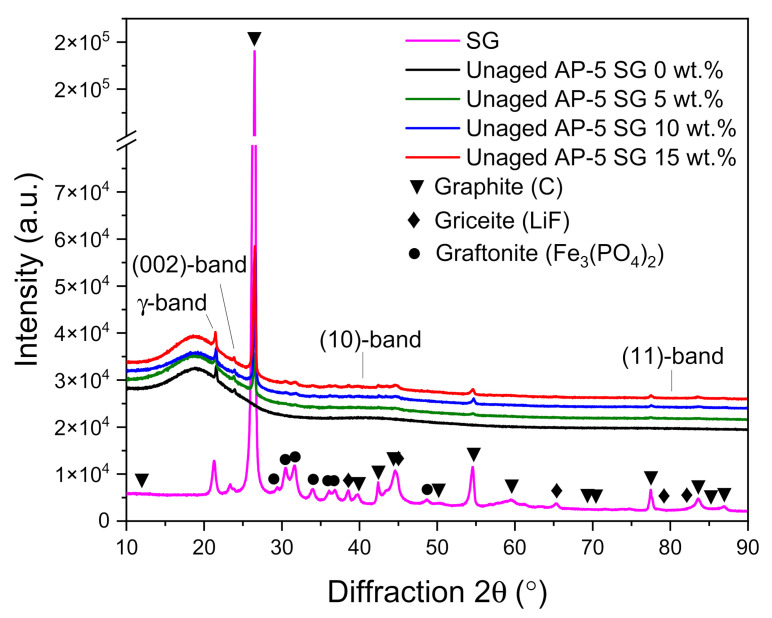
XRD diffractograms of spent graphite (SG), unmodified and base AP-5 asphalt modified with various proportions of SG (e.g., 5, 10, and 15 wt.%).

Figure 6 displays a compilation of XRD patterns divided between SG, neat asphalt, and SG-treated asphalt samples. Scanning these diffractograms, one can see that the amorphous fresh asphalt (i.e., Unaged AP-5 SG 0 wt.%) gave rise to four main characteristic bands namely: γ-band, (002)-band, (10)-band, and (11)-band. These bands arise chiefly from the crystalline asphaltene clusters embedded within the binder matrix. The γ-band or gamma band (γ) occurs as a medium-sharp and strong peak at 2θ = 21.57°. It comes mainly from condensed saturated rings and/or aliphatic chains (i.e., paraffins and naphthenes). The clear asymmetric (002)-band, which is also known as Π-band or graphene-band, settled at 2θ = 23.92° originates from the stacks of condensed aromatic sheets [68]. The two-dimensional lattice (10) and (11) reflections, or (100) and (110) reflections emerge eventually from the *in*-plane structure of aromatics. They belong to the first and second neighbors in the ring components. The (10)-band corresponds to the broad minor hump centered approximately at 2θ = 40°. This band along with the (002)-band indicates the presence of graphite-like structures (i.e., crystalline carbons) in the asphalt cement and delivers insight into the extent of condensation of the benzene rings. The (11)-band appears as a very broad peak centered at around 2θ = 80°. These broad features demonstrate that the ordering is highly dispersed in asphaltene molecules [72]. The γ-, (002)-, (10)-, and (11)-bands are common features between the base AP-5 asphalt and spent graphite (SG).

Figure 6 shows also the XRD pattern of spent graphite (SG) which reveals the existence of several peaks with different intensities laid over the 10–90° region. The first prominent tight peak falls at 2θ = 26.47° and its sharpness along with its strong signal point out that the anode-graphite possesses a very high degree of crystallinity, where the special arrangement of the crystal sheets is outstandingly arranged/organized [68].

Conspicuously, it can be seen that the SG sample comprises some impurities derived from the cathode (e.g., Graftonite crystals or Iron (II) Phosphate, Fe_3_(PO_4_)_2_ [73]) and electrolyte (e.g., Griceite or Lithium Fluoride, LiF [74]) materials.

After loading the virgin bitumen with different amounts of SG, the binder molecules are literally unable to be intercalated or inserted into the graphitic gallery space between the laminated graphene sheets due to their relatively small interlayer distances/spacings (i.e., *d*_002_ = 3.35 Å). As a result, the spent graphite (SG) will act only as a structural reinforcement material for asphalt cement and cannot produce a nanocomposite in any manner.

By delving deeper into the microstructure, it can be noticed that the introduction of 5 or 15 wt.% SG into the binder, not only amplified all the signal intensities of graphite but also slightly increased the height of all the blends’ bands and rendered them a little bit tighter. This finding endorses the fact that the carbonaceous additive expanded the crystalline phase (i.e., crystallinity or the degree of graphitization) of bitumen to some extent, which becomes more robust (i.e., stiffer) due to the further generation of asphaltenes.

The adequate cumulative impact of asphaltenes and graphite is highly expected to improve the thermomechanical properties of asphalt mixtures and prolong effectively the lifespan of road pavements.

### 3.5. Scanning Electron Microscopy (SEM)

To provide a clear picture regarding the relative effect of multiple shots of spent graphite (e.g., 5, 10, and 10 wt.% SG) on the surface texture, micromorphology, and ultimate (elemental) composition of fresh base AP-5 asphalt, scanning electron microscopy (SEM) coupled with energy-dispersive X-ray spectroscopy (EDXS) was carried out.

**Figure 7:**
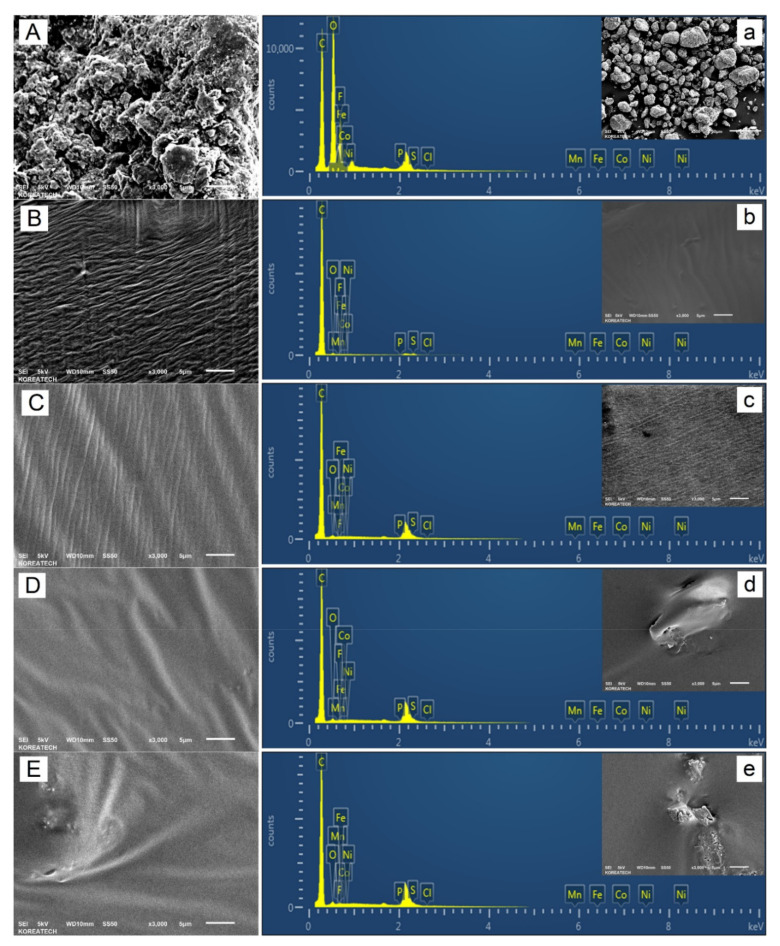
Scanning electron microscope (SEM) micrographs with their corresponding EDXS spectra of spent graphite (SG) and undosed/SG-dosed asphalt specimens taken at ×3000 magnification. (**A**,**a**) SG; (**B**,**b**) AP-5 SG 0 wt.%; (**C**,**c**) AP-5 SG 5 wt.%; (**D**,**d**) AP-5 SG 10 wt.%; and (**E**,**e**) AP-5 SG 15 wt.%.

Referring to Figure 7B,b, it can be observed that the fresh-plain bitumen presents a relatively smooth surface with uniform-wrinkled texture and homogenous structure; whereas, Figure 7A indicates that the anode-graphite specimen is characterized by a very roughened-shape morphology and sharper edges. It is mainly composed of a bulk of irregularly shaped particles sized less than 75 µm (No. 200 sieve), which are randomly widespread (Figure 7a). When mixed, this carbonaceous material will actually form a skeleton network throughout the binder and act as filling agents to confer the necessary mechanical strength to the asphalt mixtures. The rough texture and irregular shape, observed in the Figure 7A, can be related to its processing method.

Typical SEM micrographs with their corresponding EDXS analysis of asphalt samples containing several portions of spent graphite (e.g., 5, 10, and 15 wt.% SG) are portrayed in Figure 7C–E and Figure 7c–e, respectively. The element mapping from EDXS highlights that the core of spent graphite (SG) contains basically a considerable amount of carbon (C, 41.35 wt.%) and oxygen (O, 40.56 wt.%) along with some contents of fluorine (F, 16.39 wt.%) and some fingerprints of phosphorus (P, 1.70 wt.%). The richness of SG in oxygen is mainly attributed to its oxidation. The chloride (Cl), manganese (Mn), iron (Fe), cobalt (Co), and nickel (Ni) elements are undetectable. Moreover, the lithium (Li) is absent due to its small atomic number that could not be caught by SEM-EDXS scan. Initially, these findings suggest the presence of lithium salt as an electrolyte in the form of lithium hexafluorophosphate (LiPF_6_) with its products (e.g., LiF _(s)_; as confirmed by ICP-OES/FT-IR /XRD scans) and other constituents that are entrapped within the SG matrix (e.g., copper (Cu), silicon (Si), chromium (Cr), and cobalt (Co); as evinced by ED-XRFS analysis).

It is pertinent to note that the steady incorporation of SG into the original binder, increased significantly the concentration of oxygen (O) and sulfur (S) species; nevertheless, the carbon (C) value decreased fairly. The changes occurred in elemental composition reflect indeed variations in SARA fractions (i.e., a decrease in saturates, aromatics, resins contents accompanied with an increase in asphaltenes fraction).

The Figure 7C connotes that mixing the neat asphalt with 5 wt.% SG generated an even and uniform texture associated with smooth-waved surface morphology. The graphite flecks are not clearly conspicuous; however, it obvious that they are entirely embedded within the bitumen matrix, forming several conductive clusters, which may hold potential thermal-conductivity properties; responsible for improving the thermal performance of asphalt mixtures. No obvious agglomeration phenomenon was observed at the binder surface, indicating that the graphite particles may have an excellent dispersibility–compatibility with the virgin bitumen. To add further credibility to this claim further testing is highly required such utilizing Fluorescence Microscopy technique. Otherwise, the graphite nanoparticles could be successfully employed as an additive without disturbing the internal equilibrium of asphaltaneous colloidal system, which shows a typical sol-behavior (I_C AP-5 SG 5 wt.%_ = 0.5119 ≤ 0.7; stable).

When the pure asphalt is compounded with 10 wt.% SG, its topography is identified with a wide range of hills and valleys (Figure 7D) impregnated with numerous graphitic agglomerates (Figure 7d), which are seemingly dispersed satisfactorily. This scenario will actually engender a sustained absorption–adsorption of light maltene fractions (i.e., mainly saturates and aromatics) onto the high-surface area of graphite particles, resulting in an increase of asphaltenes content (i.e., from 22.38 ± 2.75 wt.% (AP-5 SG 0 wt.%) to 27.88 ± 0.87 wt.% (AP-5 SG 10 wt.%)) which will make the binder stiffer/harder and more viscous. Rising stiffness properly is expected to bring up some promising improvements on the high-temperature performance of bituminous mixtures. The initial rate of absorption is chiefly dependable on the degree of stirring, mixing time and temperature, particle-size distribution (PSD ≤ 0.75 µm) of SG, viscosity, and eventually the complex chemical nature of both asphalt and anode-graphite, and so on. Furthermore, these factors will dictate the mechanical properties of the final SG–asphalt blends.

By loading its body with 15 wt.% SG, the bitumen visually displayed a heterogeneous morphology with uneven surface microareas and irregular “hillock-like” (Figure 7E) or “mountain-like” (Figure 7e) structures. The thick and dense asphaltaneous membrane is seen to comprise some scattered islands of graphitic aggregates with different shapes and sizes (Figure 7E,e); pointing out a probable mild degree of instability–incompatibility between the spent graphite (SG) and the base AP-5 asphalt. The substantial decrease in maltene fraction after additive administration into the neat bitumen provides evidence advocating the migration of this oily-fraction from the binder into the additive. The change in binder micromorphology reflects actually changes in its colloidal system, which is tending to transit more likely from sol-like structure (I_C AP-5 SG 15 wt.%_ = 0.5587 ≤ 0.7) towards sol-gel-like structure (0.7 < I_C_ < 1.2) with further SG incorporation. If the molecular network becomes compact and more rigid, the binder will lose its ability to deform elastically. The extensive usage of waste graphite could not only damage the low-temperature performance of asphalt mixtures but also could induce the occurrence of fatigue-cracking in road pavements [75]; therefore, careful consideration should be taken.

### 3.6. Conventional Binder Tests (Penetration, Softening Point, Viscosity, and Ductility)

Conventional diagnostic methods including penetration, softening point, viscosity, and ductility were adopted to characterize the effect of various doses of spent graphite (e.g., 5, 10, and 15 wt.% SG) on the physical and rheological properties of unaged and aged (RTFO/PAV) asphalt specimens.

**Figure 8:**
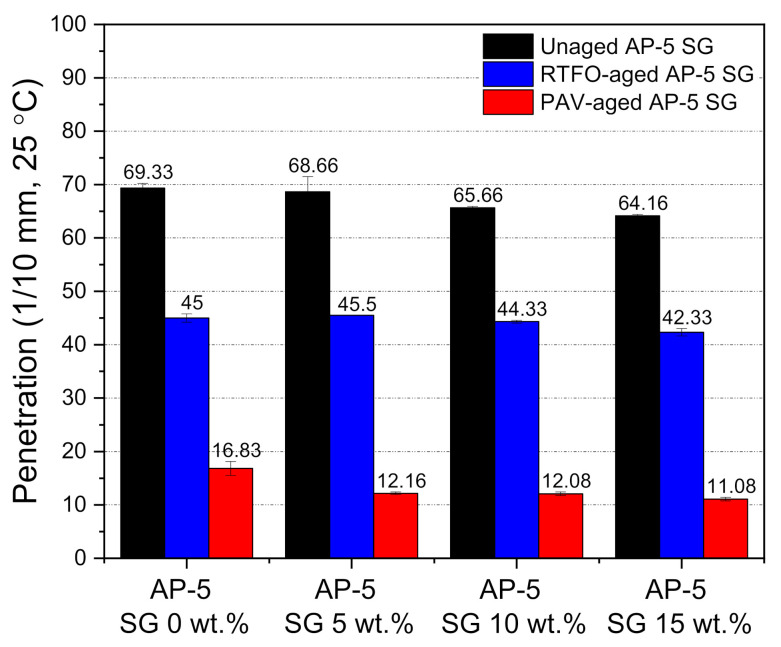
Effect of various doses of SG (e.g., 5, 10, 15 wt.%) on the penetration of base AP-5 asphalt before and after RTFO and PAV aging.

To determine the potential effect of different doses of SG on the stiffness of unconditioned and thermal-conditioned bitumen samples at intermediate temperature (ca. 25 °C), needle penetration test was executed. Referring to Figure 8, it can be clearly seen that the steady increase in SG content resulted in a decrease in penetration value for both unaged and artificial weathered bituminous specimens. This reflects that the graphite increased the binder stiffness to some extent, which in turn will enhance the rutting performance of asphalt mixtures in warm climate regions. The decline in penetration values could be related to the conversion of a great portion of free-binder into a structural-binder [20]. The binder physiochemically absorbed and/or adsorbed by the mineral powder is considered as the structural-binder; whereas, the remained portion consists the free-binder [20]. Due to the fact that the specific surface area (SSA) of spent graphite powder is considerably high, it will lead to a greater interaction between the graphite particles and the binder. Accordingly, by fortifying the base AP-5 asphalt body in SG, the volume of structural-binder will increase; meanwhile, the volume of the free one will decrease. Likewise, the interfacial adhesion strength increases with the increase in the fraction of structural-binder/polar compounds [76]; thereby resulting in a slight drop of penetration value.

In comparison to the untreated/unaged bitumen samples, the treated/aged bitumens samples noted also a decrease in their penetration values, which could be associated with a growth in the concentration of polar components rending the asphalt harder and more brittle.

**Figure 9:**
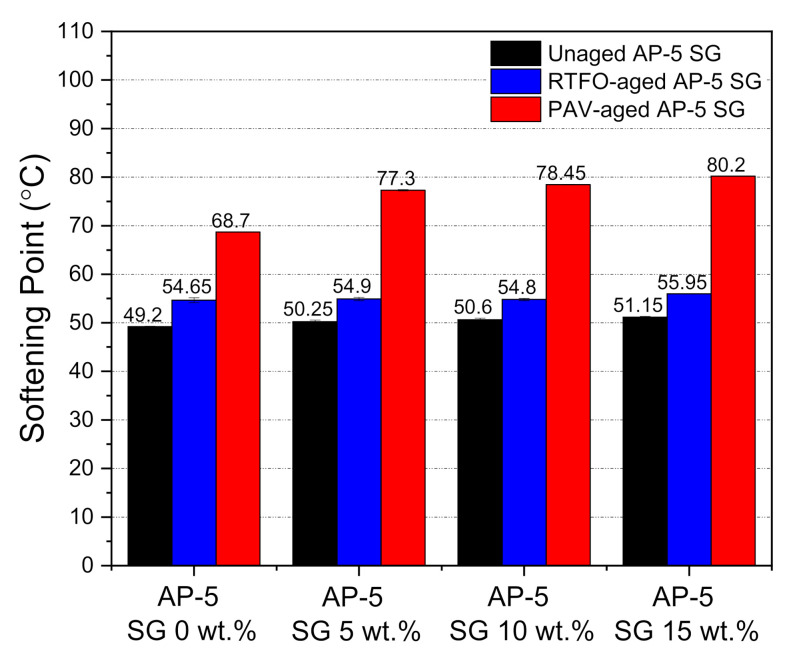
Effect of various doses of SG (e.g., 5, 10, 15 wt.%) on the softening point of base AP-5 asphalt before and after RTFO and PAV aging.

The ring-and-ball softening point technique was utilized to address the thermal attributes of unmodified and SG-modified asphalt samples prior and succeeding the aging procedures. As illustrated by Figure 9, an incremental incorporation of spent graphite (SG) into the base AP-5 asphalt increased its softening point value to some degree, before and after aging processes. This indicates that the SG modification could appreciably enhance the high-temperature properties of bitumen, thereby leading to greatly boost its anti-rutting performance. The SG–bitumen blends, which are categorized as lower-temperature susceptible binders are highly recommended to be used in warm climate zones. The sustained upsurge in T_R&B_ values is coming essentially from the structural-bitumen which apparently became more dominant than the free-bitumen following the SG treatment. With respect to plain/unaged binder, the RTFO- and PAV-aged bitumen specimens registered also a gradual growth tendency in their T_R&B_ values and this was happening due to the generation of more polar compounds, which will transfer the binder from a viscous-liquid state to an elastic semi-solid state.

**Figure 10:**
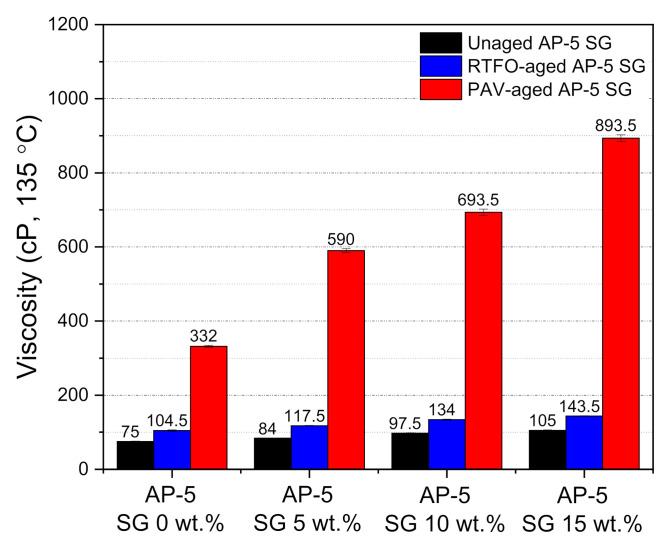
Effect of various doses of SG (e.g., 5, 10, 15 wt.%) on the viscosity of base AP-5 asphalt before and after RTFO and PAV aging.

To shed the light on the impact of spent graphite (SG) on the flowability and workability properties of unaged and aged base AP-5 asphalt molded with various proportions of SG (e.g., 5, 10, and 15 wt.%), the rotational viscosity (RV) test was conducted accordingly. As evidenced by Figure 10, the RV value noted a remarkable amplification following the incremental SG addition into the asphalt cement, rending its elastic component larger and its matrix stiffer. This should be favorable in one sense because the wettability, coating, and adhesion strength between the asphalt binder and aggregates will greatly be improved with further appropriate SG incorporation. It is worth noting that all obtained viscosity measurements are within the Strategic Highway Research Program (SHRP) specification requirements (i.e., less than 3 Pa·s at 135 °C). This performance criterion is very crucial to ensure proper pumpability, mixability, and workability of asphalt cement in the hot-mix asphalt manufacturing plant and construction site as well. After being subjected to short-term (RTFO) and long-term (PAV) aging protocols, the aged SG–bitumen samples revealed a steady trend of growth in rotational viscosity (RV) as compared to the plain unaged binder specimen. This could be linked to the oxidative aging effect, by which not only the light constituents such as saturates and aromatics are subjected to thermal degradation and evaporation (volatilization), but also the atmospheric oxygen integrates with the aromatic hydrocarbons to yield larger amounts of polar components; the major contributors to viscosity increase.

**Figure 11:**
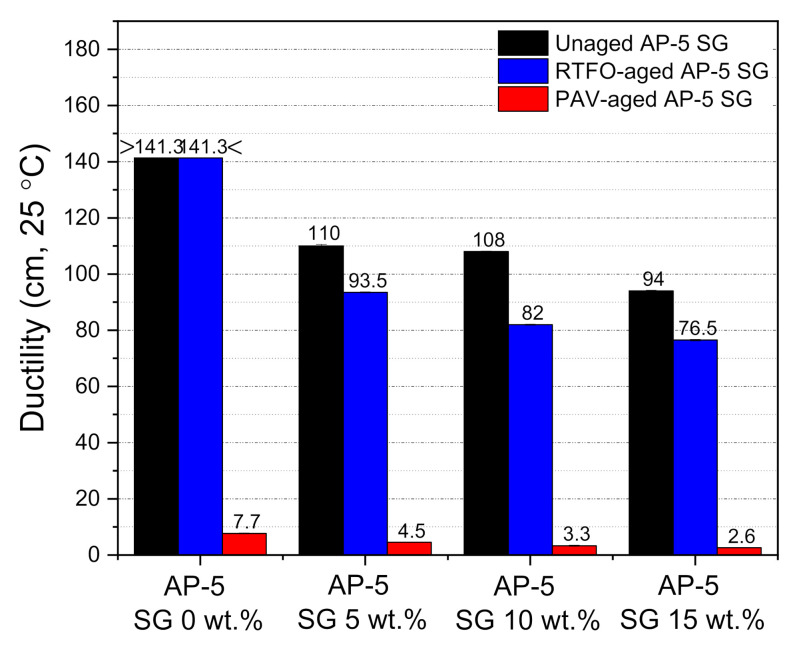
Effect of various doses of SG (e.g., 5, 10, 15 wt.%) on the ductility of base AP-5 asphalt before and after RTFO and PAV aging.

The ductility test was employed to assess the SG impact on the flexibility property and tensile deformation of base AP-5 asphalt before and after aging. The force ductility maximum load at 25 °C was recorded for unaged and aged asphalt specimens enclosing several fractions of spent graphite (e.g., 5, 10, and 15 wt.% SG). Figure 11 demonstrates that the ductility showed a trend towards diminishing, while incrementally introducing the anode–graphite powder into the binder bulk. By referring to Figure 11, it can plainly be noticed that the SG addition along with weathering treatment made the asphalt binder less ductile at 25 °C or less cohesive and stiffer. It is commonly known that the graphite is endowed with a strong oil/maltene absorption capacity, favored tremendously by its higher specific-surface area (SSA) [77,78]. This particular feature coupled with oxidative aging process, will certainly induce an instantaneous absorption of lightweight components (e.g., particularly saturates and aromatics) from the bitumen matrix; thereby contributing to its stiffening and hardening. Consequently, it can be inferred that the proper use of spent graphite (SG) from end-of-life LIBs could mitigate the impact of mechanical strains that can appear in road pavements, and thereby reducing the likelihood of rutting through the surface layer.

### 3.7. Dynamic Shear Rheometer (DSR) Test

To provide robust reasoning about the impact of various contents of spent graphite (e.g., 5, 10, and 15 wt.% SG) on the rutting potential of unblended and SG-blended asphalt samples before and after undergoing RTFO-aging, a DSR test was performed.

The deformation behavior is evaluated based on the rutting resistance factor (G*/sin δ), where |G*| is the complex shear modulus (i.e., stiffness) and δ is the phase angle (i.e., viscous δ = 90° or elastic δ = 0° indicator). A stiff and elastic binder with higher (G*/sin δ) values (i.e., less amount of energy dissipation) is strongly recommended for rutting resistance. To control the likelihood of tenderness during mixing and laying, if aging does not take place during construction, the stiffness value (G*/sin δ) of original/unaged asphalt should be higher than 1.0 kPa at the same temperature [55]. On the other side, to reduce rutting, the stiffness value (G*/sin δ) of the asphalt after RTFO should be larger than 2.2 kPa at the maximum 7-day average pavement design temperature [55].

**Figure 12:**
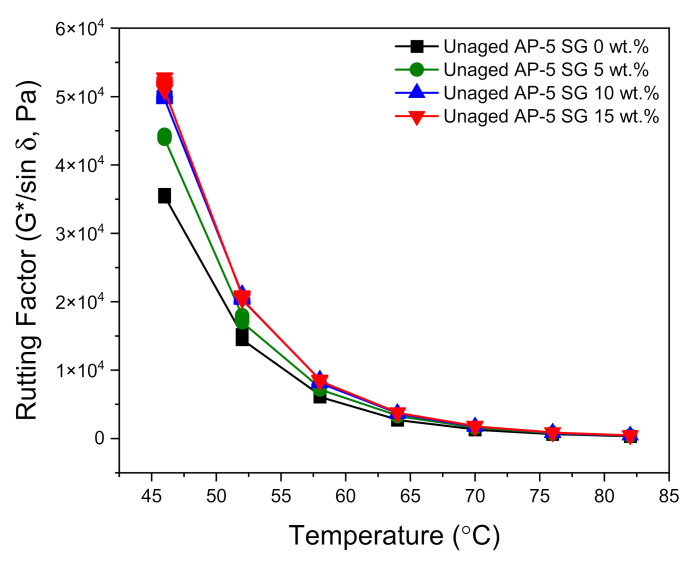
Rutting factor (G*/sin δ) versus temperature for unaged base AP-5 asphalt and its modified samples containing several proportions of SG (e.g., 5, 10, 15 wt.%).

**Figure 13:**
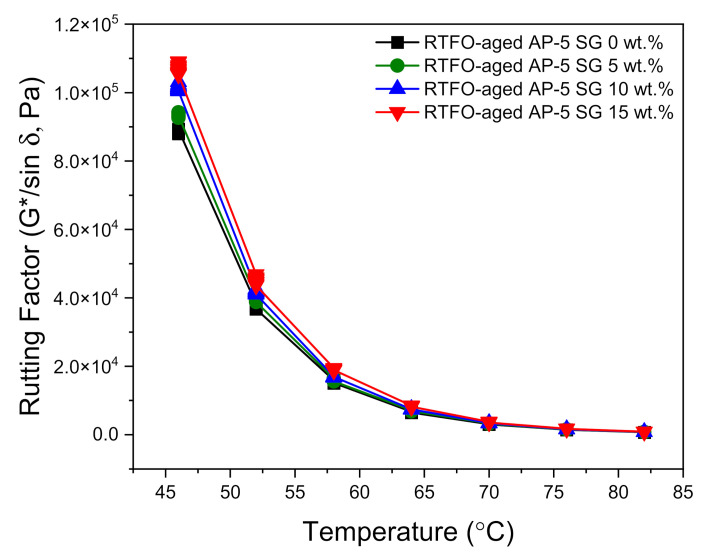
Rutting factor (G*/sin δ) versus temperature for RTFO-aged base AP-5 asphalt and its modified samples containing several proportions of SG (e.g., 5, 10, 15 wt.%).

Figure 12 and Figure 13 show the direct effects of no-conditioning and short-term thermal conditioning (RTFO) on the rutting factor parameter at higher temperatures. With regard to data plotted in Figure 12, it can be noticed that the unaged bituminous sample molded with 15 wt.% SG is endowed with the highest rutting potential as compared to the other asphaltaneous samples. Furthermore, by increasing the SG dose and testing temperature, a general trend towards increasing was established by the stiffness indicator (G*/sin δ). These findings are in a great harmony with empirical test-methods data, where the processed binders are depicted with lower penetration/ductility, higher viscosity, and softening point values. The minimum requirements (i.e., G*/sin δ ≥ 1 kPa) of Superior Performing Asphalt Pavements (Superpave) Specifications was achieved by all tested binders at temperature less than 70 °C.

Figure 13 highlights the direct influence of SG on rutting factor of short-term (RTFO) aged bituminous samples versus temperature. Similarly, the permanent deformation (i.e., rutting) output displayed an increasing tendency with increasing SG content and testing temperature. This considerable growth may reflect the formation of a graphitic skeleton network structure in the blends, which will restrain molecular association–dissociation and/or slippage motion; thereby contributing to better reinforcement of asphalt mixtures. The RTFO-aged bitumens enclosing several fractions of SG (e.g., 0, 5, 10, and 15 wt.%) are seemingly capable to sustain rutting till 70 °C (G*/sin δ ≥ 2.2 kPa). The steady increase in rutting index mirrors that the SG use is advantageous in resisting permanent deformation and could perfectly enhance the binder ability to withstand high-temperature deformation in hot seasons. The most relevant or outstanding outcome was attained by using 15 wt.% SG. Owing to its high specific surface area (SSA), high thermochemical stability, and excellent mechanical properties, the recovered graphite from dead LIBs could be used as an asphalt additive and could meaningfully ameliorate the anti-rutting performance of road pavements.

The DSR test was also carried out to determine the fatigue cracking propensity of several SG–bitumen admixtures including different percentages of SG (e.g., 5, 10, and 15 wt.%) under PAV-aged condition. The fatigue behavior is assessed based on the fatigue cracking factor (G*.sin δ). Lower values of this parameter are considered as a good indicator of fatigue cracking resistance, and vice-versa. A less stiff and more elastic binder with lower (G*.sin δ) values is highly desirable for fatigue resistance. To mitigate fatigue, the stiffness values (G*.sin δ) of the binder after the RTFO and PAV aging should be less than 5000 kPa at a specified intermediate temperature, which is equal to +4 °C plus the mean of the minimum and maximum pavement design temperatures [56]. Values beyond the 5000 kPa limit will render a pavement more prone to fatigue cracking [56].

**Figure 14:**
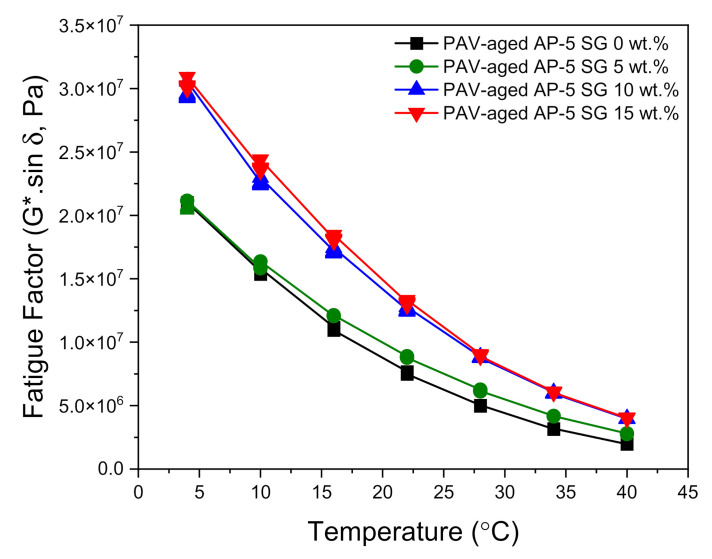
Fatigue factor (G*.sin δ) versus temperature for PAV-aged base AP-5 asphalt and its modified samples containing several proportions of SG (e.g., 5, 10, 15 wt.%).

The master curves plotted in Figure 14 reveal a strong upward trend in the fatigue cracking index (G*.sin δ) following the spiking of base AP-5 asphalt sample with multiple shots of SG. Owing to the genesis of more structural-bitumen generated by the strong interaction between the hydrophilic graphite particles and hydrophobic binder surface, the higher (G*.sin δ) values designate more shearing energy release, thereby demonstrating an inability to survive cracking stress. All PAV + RTFO-aged asphalt mixes without or with spent graphite (i.e., 0, 5, 10, and 15 wt.% SG) satisfactorily fulfilled the Superpave specification requirements (G*.sin δ ≤ 5000 kPa) at 28, 28, 34, and 34 °C, respectively, at intermediate temperatures.

Collectively, it reasonable to assume that the modification of virgin bitumen with an adequate quantity of spent graphite (i.e., <5 wt.% SG) could fortify the road pavement against extreme cracking events. However, higher doses of SG are strictly not recommended due to their harmful effects.

### 3.8. Performance Grade (PG) Test

**Figure 15:**
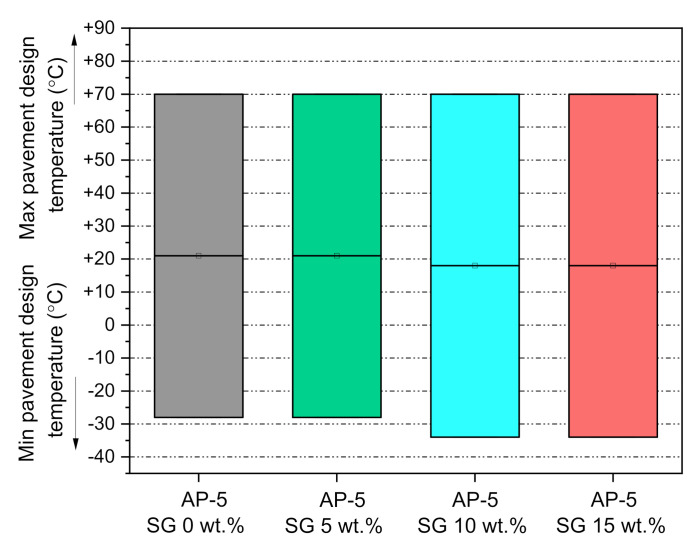
Influence of various doses of SG (e.g., 5, 10, 15 wt.%) on the performance grade (PG) of base AP-5 asphalt under unaged condition.

The Superpave Performance Classification System [79] was opted in conjunction with the several data generated by the Dynamic Shear Rheometer (DSR) to draw the diagram plotted in Figure 15. The base AP-5 asphalt understudy is classified as a PG 70-28, which means that it would exhibit a minimum failure temperature at −28 °C and a maximum failure temperature at 70 °C. The diagram displays the various changes occurring on the performance grading (PG) of fresh/neat asphalt following its treatment with different doses of spent graphite (e.g., 5, 10, and 15 wt.% SG). From Figure 15, one can easily observe that the addition of a lower concentration of SG (i.e., 5 wt.%) did not affect the PG of plain bitumen; however, the intermediate and higher concentrations of SG bumped its low-temperature performance (i.e., critical temperature for thermal cracking) downwards, by +1 below −28 °C (N.B., one grade is equivalent to +6 °C which corresponds to a doubling in the binder stiffness). Strictly speaking, the 10 and 15 wt.% dosages moderately promoted the performance of binder at low-temperatures, inasmuch as that the inferior limit of binders’ PG dropped down from −28 to −34 °C. Based on these data at hand, it can be reasonably inferred that the nanofiller employed in this investigation has improved the PG of base AP-5 asphalt at colder temperatures, without altering its high-temperature performance (i.e., critical temperature at which rutting distress cannot occur).

### 3.9. Complex Modulus (|G*|) and Phase Angle (δ)

The DSR exam was also executed to gain further insight into the impact of various quantities of spent graphite (e.g., 5, 10, and 15 wt.% SG) on the viscous–elastic balance in unaged and aged asphalt cements (i.e., base AP-5 asphalt) at medium to high temperatures.

The complex shear modulus |G*| designates the total overall resistance of asphalt sample against a constant shear-deformation; meanwhile, the phase angle (δ) denotes the time-lag between the maximum applied shear-stress and the maximum resulting shear-strain response [80,81]. Generally, a stiffer binder is associated with higher |G*| value and greater potential for cracking. On the other side, a higher viscous binder is accompanied with higher δ value. Commonly, the δ values are swinging between 0° and 90°. When a given asphaltaneous material reaches 0°, it becomes purely elastic and converts into purely viscous–plastic state when it attains 90° [80,81]. Traditionally, conventional paving bitumens are depicted with |G*| values ranging between 0.08 and 0.87 psi (i.e., 500 to 6000 Pa) and δ values stretching from 50° to 90° [80,81]. The two main viscoelastic parameters (|G*|, δ) are largely dependable on certain factors including but not limited to bitumen performance grade (PG), climatic conditions, traffic loads, structural design, and experimental conditions such as temperature, loading time and frequency, etc.

**Figure 16:**
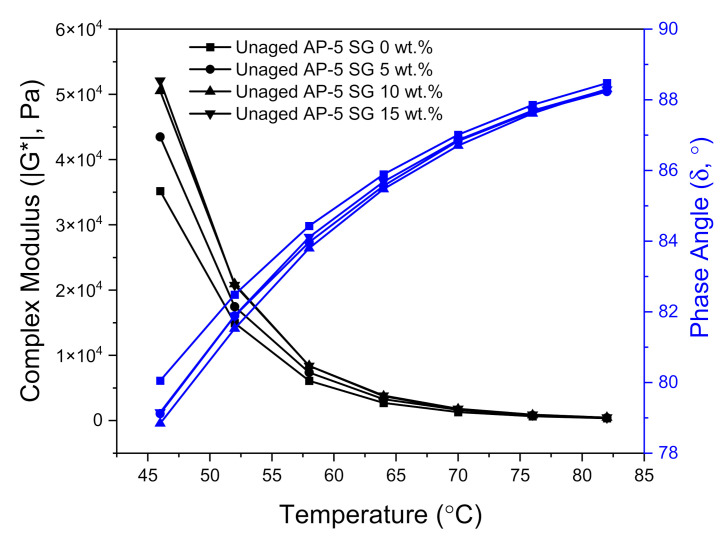
Variation of complex modulus (|G*|) and phase angle (δ) as a function of temperature for unaged base AP-5 asphalt and its samples treated with various doses of SG (e.g., 5, 10, 15 wt.%).

**Figure 17:**
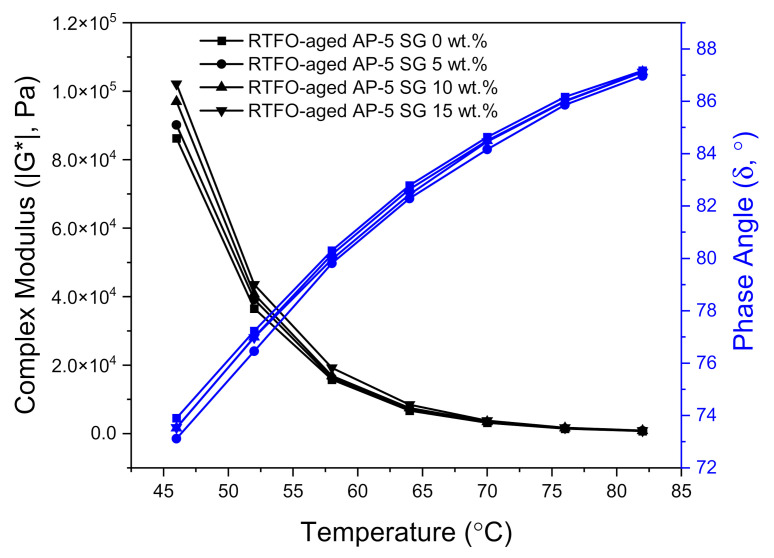
Variation of complex modulus (|G*|) and phase angle (δ) as a function of temperature for RTFO-aged base AP-5 asphalt and its samples treated with various doses of SG (e.g., 5, 10, 15 wt.%).

Figure 16 and Figure 17 illustrate the variation of complex modulus (|G*|) and phase angle (δ) of unmodified and SG-modified asphalt samples at higher temperatures and a constant frequency of 10 rad s^−1^, under unaged and RTFO-aged conditions, respectively.

In reviewing Figure 16 and Figure 17, it can be clearly seen that with temperature elevation, the complex modulus |G*| noted a steady decrease; whereas, the phase angle (δ) recorded a gradual increase for all unaged asphalt binders. Actually, these changes took place as a normal result to the innate rheological behaviors of asphalt binder. When the temperature augments, the viscous component greatly expands and becomes more dominant than the elastic one. To put it in simple terms, when the binder underwent a consistent thermal treatment, its free volume will show high dilatation tendency and its high elastic-state at low temperatures will be transformed into a viscous-flow state at elevated temperatures, resulting in reducing the shears tress of bitumen as well as in increasing its shear strain.

The unaged and RTFO-aged AP-5 asphalt along with its specimens containing diverse fractions of SG (e.g., 5, 10, and 15 wt.%), behaved similar to a viscous liquid–fluid and marked a constant upsurge in |G*| values associated with a slight decrease in δ values as the additive concentration and temperature increased together, pointing out the positive impact of SG in the elevated temperature range. These findings underscore the fact that the rigid-crystalline structure of SG made the base AP-5 asphalt stiffer and more robust, thereby improving its deformation resistance under shear loadings. Dissimilarly, the phase angles in conjunction with complex shear moduli of SG–bitumen blends faced a moderate reduction following the SG treatment. This reflects that the stepwise addition of graphite powder into the binder had an impact on its viscous response, which decreased minorly, rather than the elastic response at all testing temperatures.

**Figure 18:**
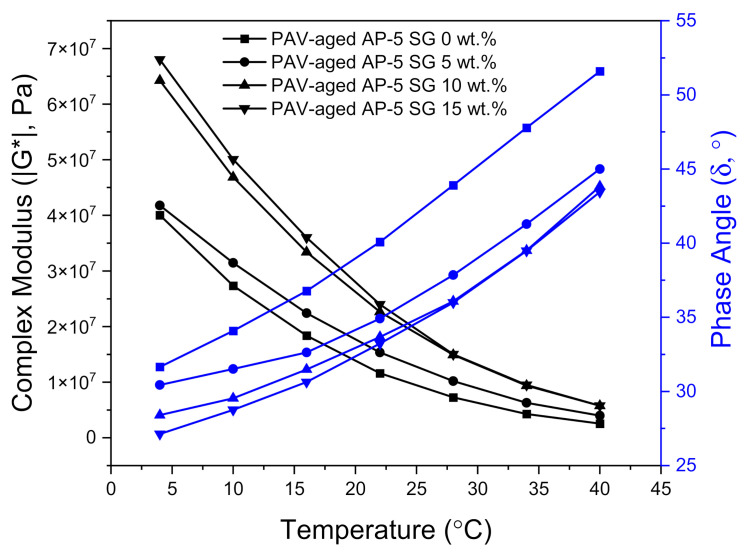
Variation of complex modulus (|G*|) and phase angle (δ) as a function of temperature for PAV-aged base AP-5 asphalt and its samples treated with various doses of SG (e.g., 5, 10, 15 wt.%).

The effects induced by long-term (PAV) aging process on the viscoelastic characteristics of undosed and SG-dosed asphalt specimens are plotted in Figure 18. Upon administering multiple SG doses into the neat bitumen body, the PAV-aging unequivocally generated a shift of the master curves towards higher complex shear moduli (i.e., upward shift) and lower phase angles (i.e., downward shift), thus revealing a noticeable change in rheological behavior consisting essentially in a steady increase of stiffness modulus (i.e., rigidity) coupled with a gradual increase in elastic response.

From the foregoing data, it is evident that that the spent graphite (SG) extracted from retired LIBs is well equipped with a huge potential to boost anti-rutting performance at medium and high temperatures.

### 3.10. Multiple Stress-Creep Recovery (MSCR) Test

The MSCR test applied at higher temperatures is strongly effective in assessing the stress dependency and rutting performance of bitumen since it mimics the real loading conditions for road pavements. To investigate in greater depth the impact of various dosages of spent graphite (e.g., 5, 10, and 15 wt.% SG) on the rutting performance of the RTFO-aged bitumen sample (i.e., base AP-5 asphalt), an MSCR test was conducted by employing DSR.

**Figure 19:**
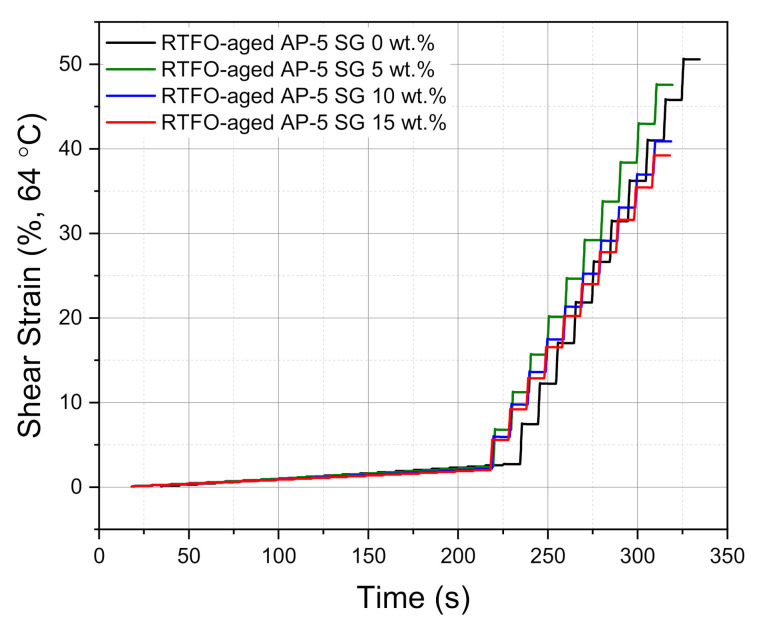
Multiple stress-creep recovery (MSCR) test results, achieved at 0.1 kPa, 3.2 kPa, and 64 °C, for RTFO-aged base AP-5 asphalt and its specimens comprising various fractions of SG (e.g., 5, 10, 15 wt.%).

Figure 19 summarizes the MSCR data obtained at two stress levels: 0.1 kPa and 3.2 kPa. The first stress level 0.1 kPa is opted because it represents the asphalt binder behavior within the linear viscoelastic area; whilst, the second stress level 3.2 kPa is selected since it expresses the binder behavior within the non-linear viscoelastic area [82].

Typically, when the short-term aged binder is characterized by a greater percentage of recoverable strains (*R%*), it means it holds higher anti-rutting potential [63]. Referring to the virgin binder, the blend enclosing 5 and 10 wt.% SG exhibited the highest *R%* value, thereby indicating that the incremental incorporation of mineral-additive into the binder could enhance the resistance of asphalt mixtures against permanent deformation and hence the high-temperature performance. Such improvement can be justified by the creation of crosslinked graphitic network structure throughout the binder matrix, resulting in yielding more structural-binder and subsequently delivering better rutting performance.

Based on the data compiled in Table 3, it can be readily observed that the SG-modified asphalt specimens are identified with lower non-recoverable creep compliance (*J_nr_*) values as compared to the *J_nr_* value of plain bitumen. This indicates that all bituminous samples containing several fractions of SG are less prone to rutting or shear failures. At this level, it can be argued that the base AP-5 asphalt mixed with 15 wt.% SG is highly expected to offer superior performance in terms of strength and durability.

In reviewing the AASHTO M 332 Standard Specification [59], the performance requirements of bitumen against rutting were detailly described by applying the MSCR test and adopting *J_nr_* at a stress 3.2 kPa level. It is stated that *J_nr_*_3.2_ ≤ 4.0 kPa^−1^ is accounting for standard traffic (S) loading, *J_nr_*_3.2_ ≤ 2.0 kPa^−1^ for heavy traffic (H) loading, *J_nr_*_3.2_ ≤ 1.0 kPa^−1^ for very heavy traffic (V) loading, and *J_nr_*_3.2_ ≤ 0.5 kPa^−1^ for extremely heavily traffic (E) loading. A cursory glance at the *J_nr_*_3.2_ values condensed in Table 3 demonstrates that the plain bitumen along with its admixtures containing 5, 10, and 15 wt.% SG could bear (H) loading, versus rutting at 64 °C.

Even though the Superpave Performance Grading (PG) defines the principal requirement for *J_nr_* at 3.2 kPa, the shear stress 0.1 kPa plays also an extremely important role in the defense of some unduly vulnerable asphalt binders against shear stress change and densification (i.e., volume variation). Accordingly, the AASHTO M 332 [59] introduced another paramount and stringent requirement necessitating that the difference in *J_nr_* values between 0.1 kPa and 3.2 kPa should be less than 75%. Taking a closer inspection of Table 3, it can be noticed that there is not any SG–bitumen blend including SG that carries a ∆*J_nr_* ≥ 75%, thereby expressing lower sensitivity towards shear stress oscillation. However, all tested bituminous specimens such as the virgin base AP-5 asphalt along with its specimens molded with 5, 10, and 15 wt.% SG displayed lower ∆*J_nr_* percentages. This observation vindicates that, lower, intermediate, and higher doses of spent graphite have no marked influence on the susceptibility of non-recoverable creep compliance (*J_nr_*) in response to the shear stress deviations. In other words, when challenged with either unpredicted heavy loads or exposed to high temperatures, the concerned binders are unlikely to experience substantial strains.

The variation of accumulated strain (%) versus time (s) at 0.1 kPa and 3.2 kPa, at an ordinary high-operating temperature of 64 °C, is plotted in Figure 19. Careful visual examination of this figure indicates that the carbonaceous additive affected drastically the creep and recovery parameters. After undergoing multiple creep-stress cycles, the plain binder embedded with higher content of recovered graphite (i.e., 15 wt.% SG) manifests the lowest accumulated strain as compared to the other asphaltaneous specimens. In the light of these findings, it could be confirmed that the SG addition has a promising impact on the permanent deformation and bleeding resistance of asphalt mixtures.

### 3.11. Merits and Demerits of Laboratory Techniques

The different tools employed to investigate the effect of spent graphite (SG) on the microstructural, morphological, physicochemical, and rheological properties of base AP-5 asphalt are diverse and each is able to deliver promising data. While, it is crucial to comprehend the abilities of each technique [83,84,85,86,87], it is also vitally important to gain deeper insights into their limitations [83,84,85,86,87]. For instances:

− The TLC-FID technique can offer a quick information about the influence of SG on the chemical composition of base AP-5 bitumen, while separating, identifying, and quantifying the various chemical species making up the filler as well as the asphaltic mastics. Iatroscan analysis requires smaller amounts of samples or solvents, but it is mostly lacking quantitative accuracy and reproducibility.
− The FT-IR spectroscopy can provide some useful knowledge regarding the most remarkable alterations and changes that have occurred at the molecular level of asphalt cement, following its blending with numerous SG fractions. On the other side, this qualitative method cannot gain access into the elemental content of bituminous specimens.
− The XRD spectroscopy is a fast and non-destructive technique that can effectively elucidate the SG impact on the microphase and microcrystalline structure of asphalt binder, while identifying several mineral elements embedded within the SG–bitumen matrix. The XRD suffers from size limitations. In fact, it cannot detect elements with smaller structures and existing in fingerprint amounts, which can generate skewed results. Furthermore, different compounds could produce similar XRD patterns.
− The SEM-EDXS is a relatively inexpensive, rapid, and effective analytical technique that can provide detailed information related to the topography, micromorphology, and ultimate composition of SG-treated asphalt samples. However, it could be destructive and could thermally damage the bituminous films during scanning.
− The empirical test methods (e.g., penetration, softening point, viscosity, and ductility) can provide some information regarding the SG influence on the physical properties of asphalt cement, but due to their empirical nature they cannot directly be correlated to the required rheological performance of asphalt and the results cannot be expressed in engineering units. Moreover, by adopting different conditions (e.g., T°C, time, loading modes and rates, etc.), some misleading conclusions could be drawn.
− The DSR test enables the measurements of a wide range of parameters including but not limited to the performance grade (PG), complex shear modulus |G*|, phase angle (δ), rutting factor (G*/sin δ), and fatigue cracking factor (G*.sin δ). Nevertheless, the obtained results could be relatively affected by several factors viz.: (1) sample preparation, (2) sample geometry, (3) temperature control, (4) linear viscoelasticity, and (5) equilibrium calibration, etc.

In order to obtain accurate, reliable, repeatable, and reproducible data, it is highly advisable to learn in-depth about the use, advantages, and drawbacks of any laboratory technique before proceeding with experiments. Additionally, by carefully studying and thoroughly understanding the nature and the properties of the material under investigation in conjunction with the multiple internal and external factors that can be involved during analysis, valuable findings could be achieved as those reported in this work.

## 4. Conclusions

In this research project, the effect of various portions of spent graphite (SG) recovered from retired lithium-ion batteries (LIBs) on the physicochemical and rheological properties of base AP-5 asphalt without or with aging (e.g., RTFO and PAV) was extensively investigated. The multiple benefits resulting from combining SG with asphalt cement worked together to generate a superior performance bituminous product. Under unaged conditions, TLC-FID (Iatroscan) analysis showed that the SG treatment, generally reduced the fractions of saturates, aromatics, and resins, and increased the content of asphaltenes. Nevertheless, under aged conditions, the aliphatic (i.e., saturates), aromatics, and asphaltenes compounds displayed a declining trend associated with a significant growth in naphthenic hydrocarbons (i.e., resins). Consequently, the stable–colloidal system of bitumen exhibited a transition tendency from sol- towards sol-gel character. FT-IR scan established that the SG is chemically inert rather than active. Its filling effect lowered the rate of oxidation and age-hardening of asphalt binder. XRD substantiated that the steady SG addition into the binder created a lattice structure, which holds a stiffening effect, thereby increasing the crystallinity of the resultant mastics. Thorough examination of SEM micrographs evinced that the lower proportion of spent graphite (i.e., 5 wt.% SG) did not significantly alter the overall morphology of bituminous mastics; thereby indicating, a larger degree of compatibility and dispersibility within the binder matrix. However, the intermediate and higher doses (i.e., 10 and 15 wt.% SG) made the binder microsurface relatively rougher. On one side, the SG enhanced the stiffness, softening point, and viscosity of the asphalt binder. On the other side, it adversely impacted its ductility. DSR test demonstrated that the carbonaceous additive improved the low-temperature performance grade (PG) of the base AP-5 asphalt and rendered it more resistive to rutting but did not provide enough fracture toughness at intermediate temperatures. MSCR test endorsed that lower non-recoverable creep compliance (*J_nr_*) along with higher percent recovery (*R%*) can be achieved by using SG; thereby imparting the bitumen with excellent deformation resistance. Conclusively, the SG has shown great promise to be used either as an additive or filler towards enhancing the road pavement performance, while alleviating the pollution generated by decommissioned LIBs.

For future work, it is strongly recommended to conduct comparative studies on the influence of different types of graphite (e.g., commercial, unpurified (this work), and purified) on the attributes of AP-5 bitumen. Additional test methods may involve the use of Bending Beam Rheometer Test (BBRT), Moisture Susceptibility Test (MST), Direct Tensile Test (DTT), Marshall Stability and Flow Test, Wheel-Tracking Test, Adhesion Strength Test, etc.

Finally, all the developments planned in this research project will be accurately analyzed through Health Risk Assessment (HRA), Life Cycle Analysis (LCA), and Life Cycle Cost Analysis (LCCA), which will demonstrate the socio-economic and environmental benefits of the solution proposed apart from contributing of the technology and the revelation of its advantages to the stakeholders.

## Figures and Tables

**Table 1:** Chemical characteristics of spent graphite (SG).

Elemental Analysis ^(a)^	Mean ± SD
C (Carbon)	68.86 ± 4.02 wt.%
H (Hydrogen)	0.47 ± 0.02 wt.%
N (Nitrogen)	0.43 ± 0.04 wt.%
S (Sulfur)	0.00 ± 0.00 wt.%
O (Oxygen)	14.19 ± 0.04 wt.%
**Elemental Analysis** ^(b)^	
Cu (Copper)	86.13 ± 0.06 wt.%
P (Phosphorus)	12.17 ± 0.03 wt.%
S (Sulfur)	1.10 ± 0.01 wt.%
Si (Silicon)	0.40 ± 0.02 wt.%
Cr (Chromium)	0.10 ± 0.00 wt.%
Co (Cobalt)	0.07 ± 0.00 wt.%
**Elemental Analysis** ^(c)^	
Li (Lithium)	47.42 ± 0.01 mg g^−^^1^
**SARA Generic Fractions**	
Saturates	5.32 ± 0.82 wt.%
Aromatics	2.50 ± 0.22 wt.%
Resins	4.00 ± 0.57 wt.%
Asphaltenes	88.16 ± 1.02 wt.%

^(a)^ EA Flash 2000; ^(b)^ ED-XRFS EDX-720; ^(c)^ ICP-OES iCAP 6000 Series.

**Table 2:** Physicochemical properties of base AP-5 asphalt.

Elemental Analysis ^(a)^	Mean ± SD
C (Carbon)	83.53 ± 0.08 wt.%
H (Hydrogen)	9.88 ± 0.03 wt.%
N (Nitrogen)	0.92 ± 0.02 wt.%
S (Sulfur)	4.38 ± 0.24 wt.%
O (Oxygen)	0.59 ± 0.06 wt.%
**SARA Generic Fractions**	
Saturates	6.63 ± 1.00 wt.%
Aromatics	15.18 ± 2.20 wt.%
Resins	55.78 ± 1.44 wt.%
Asphaltenes	22.38 ± 2.75 wt.%
**Physical Properties**	
Penetration at 25 °C	69.33 ± 0.93 dmm
Softening point	49.20 ± 0.11 °C
Rotational viscosity at 135 °C	75 ± 1.06 cP
Ductility at 25 °C	>141.30 ± 00 cm

^(a)^ EA Flash 2000.

**Table 3:** Multiple stress-creep recovery (MSCR) parameters, obtained at 0.1 kPa, 3.2 kPa, and 64 °C, for RTFO-aged base AP-5 asphalt and its samples containing various doses of SG (e.g., 5, 10, 15 wt.%).

Asphalt Binder Type	MSCR Data at 64 °C					
	*R*_0.1_ (%)	*R*_3.2_ (%)	*J_nr_*_0.1_ (kPa^–1^)	*J_nr_*_3.2_ (kPa^−1^)	∆*J_nr_* (%)	PG Grade
AP-5 SG 0 wt.%	−4.20	−8.00	1.3558	1.4959	10.30	PG 64 H
AP-5 SG 5 wt.%	−4.00	−7.80	1.2104	1.4124	16.70	PG 64 H
AP-5 SG 10 wt.%	−4.10	−7.70	1.1026	1.2103	9.80	PG 64 H
AP-5 SG 15 wt.%	−4.20	−7.90	0.9982	1.1631	16.50	PG 64 H

## Data Availability

The data presented in this study are available on request from the corresponding author.
